# Effect of Experimental Electrical and Biological Parameters on Gene Transfer by Electroporation: A Systematic Review and Meta-Analysis

**DOI:** 10.3390/pharmaceutics14122700

**Published:** 2022-12-02

**Authors:** Tjaša Potočnik, Alenka Maček Lebar, Špela Kos, Matej Reberšek, Eva Pirc, Gregor Serša, Damijan Miklavčič

**Affiliations:** 1 Faculty of Electrical Engineering, University of Ljubljana, Tržaška 25, 1000 Ljubljana, Slovenia; 2 Department of Experimental Oncology, Institute of Oncology Ljubljana, Zaloška cesta 2, 1000 Ljubljana, Slovenia

**Keywords:** gene electrotransfer, plasmid DNA, nucleic acid, pulse parameters, electroporation medium, cell line

## Abstract

The exact mechanisms of nucleic acid (NA) delivery with gene electrotransfer (GET) are still unknown, which represents a limitation for its broader use. Further, not knowing the effects that different experimental electrical and biological parameters have on GET additionally hinders GET optimization, resulting in the majority of research being performed using a trial-and-error approach. To explore the current state of knowledge, we conducted a systematic literature review of GET papers in in vitro conditions and performed meta-analyses of the reported GET efficiency. For now, there is no universal GET strategy that would be appropriate for all experimental aims. Apart from the availability of the required electroporation device and electrodes, the choice of an optimal GET approach depends on parameters such as the electroporation medium; type and origin of cells; and the size, concentration, promoter, and type of the NA to be transfected. Equally important are appropriate controls and the measurement or evaluation of the output pulses to allow a fair and unbiased evaluation of the experimental results. Since many experimental electrical and biological parameters can affect GET, it is important that all used parameters are adequately reported to enable the comparison of results, as well as potentially faster and more efficient experiment planning and optimization.

## 1. Introduction

Transfection, a method in which foreign nucleic acids (NAs) (DNA, RNA) are introduced into cells to produce genetically modified cells, is an indispensable method in basic genetic research, bioproduction, gene therapy, cell therapy, and vaccine approaches. Since it was demonstrated in 1982 that naked NAs could be successfully transferred into cells using high-voltage electric pulses [1], the method, now known as gene electrotransfer (GET), has received considerable attention. It is relatively inexpensive, flexible, and safe for clinical use. Moreover, it can be used to treat many individual cells within a short time in in vitro conditions, and it delivers naked pDNA into cells without the use of additional chemicals or viruses. GET increases plasmid DNA (pDNA) transfection rates by 100–2000-fold and improves transfection reproducibility compared with injecting pDNA without applying electric pulses [2]. However, because the mechanisms of GET in cells [3] and the role of experimental electrical and biological influencing parameters are still unclear [4], the efficiency of GET could still be improved. Electrical parameters are determined by the choice of generator of the electric pulses, also named the electroporator, and the electric pulse parameters, as well as by the geometry and material of the electrodes [5,6]. Biological parameters include the biological properties of the cells or tissue [7], the biological and physical properties of the extracellular environment [3,8,9], and the properties and amount of the used NA [10]. Finally, when GET efficiency is measured, the methods used to determine the GET efficiency and the time at which GET is evaluated are also important.

GET is achieved when NAs enter the cell and produce a protein or shut down a defective or overexpressed gene. GET is most often used to deliver pDNA, but in recent years, different RNAs have also been delivered by electroporation. Depending on the type and size of NAs introduced into cells, they must cross several barriers before reaching the cytoplasm of the cell (in the case of RNA) or the cell nucleus (in the case of DNA) to exert their therapeutic effect [2]. GET is a multistep process that includes, in the case of plasmid DNA (pDNA), interaction of pDNA with the cell membrane, translocation across the cell membrane, migration through the cytoplasm, translocation through the nuclear envelope, and gene expression [3]. At GET, the electric field present when electric pulses are applied to cells allows the permeabilization of the cell membrane and electrophoresis of negatively charged NA molecules from the cathode to the anode, bringing them into contact with the cell membrane in greater numbers compared to free diffusion [11]. Larger pDNA molecules (greater than 1 kb) interact with the cell membrane and form aggregates on the cell membrane during pulse delivery [12]. These pDNA aggregates must then be transferred into the cell, transported intracellularly through the cytoplasm, and imported through the nuclear envelope into the nucleus [3,13]. Endocytosis and cell membrane repair mechanisms following the delivery of electric pulses have been suggested as a possible mechanism for pDNA internalization [2,14]. Small NA molecules enter the cell mainly by electrophoresis during pulse delivery through permeable sites in the cell membrane. For RNA molecules, it was shown that they enter the cell through permeable sites of cell membrane during pulse delivery without forming aggregates on cell membrane, and thus have direct access to cell cytoplasm [15]. Still, the exact mechanism enabling different NAs to cross the abovementioned barriers is largely unknown. 

The electric field leading to cell membrane permeabilization, a crucial step of GET, is governed by the electrode geometry, dielectric properties of the load (i.e., cells in vitro or tissue in vivo), and the amplitude of the electric pulses delivered by the electroporator. In vitro, two-plate electrodes with a spacing of at least 1 mm (i.e., macroelectrodes) are most often used, where the cells are exposed to the electric field. They are very commonly provided in the form of commercially available cuvettes. Other types of electrodes include single-cell chambers, microelectrodes (glued to cover glass), and flow-through chambers (i.e., polyethylene or polypropylene as an insulating material combined with stainless steel electrodes) [16,17]. Not only the geometry but also the material of the electrodes is important because electrochemical reactions occurring at the electrode-electrolyte interface during the delivery of high-voltage electric pulses. Electrolysis, the formation of radicals, and the release of metal ions from the electrodes lead to the corrosion and contamination of the electrodes, as well as the chemical alteration of the medium [8,9,18,19].

The parameters of electric pulses are the pulse shape, amplitude and duration, number of pulses, pulse repetition frequency, and polarity [16]. The exponentially decaying pulses originally used have been largely replaced by GET protocols that favor the use of long monopolar millisecond pulses, which provide sufficient electrophoresis to bring a sufficient number of NA molecules into contact with the cell membrane [3,11]. However, long monopolar pulses also cause considerable electrode oxidation, pH changes near the electrodes, bubble formation, and Joule heating [18,20,21,22]. All these phenomena are undesirable in GET applications. In applications in vivo, the additional disadvantages are discomfort and pain, requiring the use of muscle relaxants, anesthesia, and synchronization of pulse delivery with the electrocardiogram [23]. The use of high-frequency bipolar pulses (HF-BP) and nanosecond pulses (ns) [11] reduces electrochemical reactions [18], muscle contractions, and pain [23,24,25,26]. In an attempt to improve the efficiency of GET, various combinations of millisecond, microsecond, and nanosecond pulses have also been used [27,28,29]. 

Biological characteristics of the cells and the extracellular environment, such as the composition of the cell membrane, the conductivity of the medium surrounding the cells [30,31,32] and its pH [33], the volume fraction of the cells, and the arrangement of the cells in the suspension [34,35] can also affect the efficiency of GET [3,8,9,33]. Different papers have reported that GET is highly cell line-dependent, with primary cells usually being harder to transfect compared to normal and tumor cell lines [11,36].

In vitro GET is often performed in a special electroporation media of low conductivity to reduce the power requirements of the electroporation device. In vitro electroporation is also often performed in a cell growth medium, which is an approximation of tissue intracellular fluid [11,37]. However, the cell growth medium consists of many components which can affect cell membrane permeabilization and, consequently, GET. 

GET efficiency also depends on NA properties such as topology, size, concentration, and mobility. Smaller pDNA molecules have been shown to lead to better GET efficiency, presumably due to their higher mobility [32]. Higher pDNA concentration also resulted in higher GET efficiency, especially when shorter (i.e., less than 10 µs) pulses were used, but have led to a decrease in cell survival in some cell lines [11,37,38]. In the case of DNA molecules, the promoter used can also affect the dynamics of transgene transcription and, consequently, GET efficiency [39]. 

The fact that the exact mechanisms of NA delivery with GET are still unknow represents a limitation for its broader use. Further, not knowing the effects that different experimental electrical and biological parameters have on GET additionally hinders GET optimization. As a result, the majority of research is performed using a trial-and-error approach, which is not always the most successful. In addition, a trial-and-error approach results in a large number of papers. To explore the current state of knowledge on the influence of electrical and biological parameters affecting the efficiency of GET, we conducted a systematic literature review of GET papers in in vitro conditions and performed meta-analyses of the reported GET efficiency. To our knowledge, this is the first systematic review that summarizes all influential parameters considered in reporting GET efficiency. We believe that the results of this study will improve our understanding of GET and provide guidance for future study reports.

## 2. Materials and Methods

### 2.1. Eligibility Criteria

Only original research, peer-reviewed in vitro papers based on eukaryotic cells were included in the review. Theoretical papers (modeling), in vivo, in ovo and clinical studies, papers reporting GET on prokaryotes, papers in which GET was used only as a tool, papers that did not test multiple electroporation parameters, and papers with only “nucleofection” were excluded, as no parameters were reported that could be used in our analysis. 

### 2.2. Search Strategy

A comprehensive electronic search was carried out in the Scopus, Web of Science, and PubMed databases to identify eligible papers in English language only. The initial search was performed in August 2018 and repeated in May 2021 and July 2022. The following search terms/words were used: 

Scopus: (ALL (gene AND transfer OR DNA AND vaccination OR plasmid AND DNA) AND ALL (electroporation) AND ALL (optimi* OR compar* OR multiple AND parameter*)) AND (LIMIT-TO (DOCTYPE, “ar”)) AND (LIMIT-TO (LANGUAGE, “English”)).

(ALL (gene AND electrotransfer OR electrogene AND transfer) AND ALL (optimi* OR compar* OR multiple AND parameter*)) AND (LIMIT-TO (DOCTYPE, “ar”)) AND (LIMIT-TO (LANGUAGE, “English”)).

Web of Science: TOPIC: (gene transfer OR DNA vaccination OR plasmid DNA) AND TOPIC: (electroporation) AND TOPIC: (optimi* OR compar* OR multiple parameter*) Refined by: LANGUAGES: (ENGLISH) AND DOCUMENT TYPES: (ARTICLE) Timespan: All years. Databases: WOS, BCI, CCC, DRCI, DIIDW, KJD, MEDLINE, RSCI, SCIELO, ZOOREC. Search language = Auto.

TOPIC: (gene electrotransfer OR electrogene transfer) AND TOPIC: (optimi* OR compar* OR multiple parameter*) Refined by: LANGUAGES: (ENGLISH) AND DOCUMENT TYPES: (ARTICLE) Timespan: All years. Databases: WOS, BCI, CCC, DRCI, DIIDW, KJD, MEDLINE, RSCI, SCIELO, ZOOREC.

PubMed: Search (gene transfer OR DNA vaccination OR plasmid DNA) AND electroporation AND (optimi* OR compar* OR multiple parameter*) Filters: Full text; English.

Search (gene electrotransfer OR electrogene transfer) AND (optimi* OR compar* OR multiple parameter*) Filters: Full text; English.

### 2.3. Paper Selection

The first screening of potentially relevant papers was performed based on the title and abstract. At this stage, papers that did not report GET or reported GET on prokaryotes, in vivo, in ovo, clinical, and theoretical papers were excluded. We also excluded papers in which GET was used only as a tool and thus did not test multiple electroporation parameters, and papers in which only “nucleofection” was used. After initial screening, full-text versions from the papers that compared GET efficiency depending on varying at least one electrical or biological parameter were obtained if possible. 

Since many abbreviations are used throughout the paper, we summarized them all in Table 1.

### 2.4. Data Extraction

Bibliographic details of the papers, such as authors, journal, and year of publication, were retrieved.

Electrical parameters: The type of electrodes, material of the electrodes, device used to apply electric pulses (type and producer), amplitude and duration of electric pulses, and potential measurement of output pulses were collected. 

The types of electrodes were further divided into six groups: plate, wire, neon tip, electroporation cuvette, custom-made electrodes, and not reported. All types of electrodes that did not match the first four groups were classified into a custom-made electrodes group. If the study reported that electroporation cuvettes were used and the material of the electrodes was not reported, then we determined the material of the electrodes based on the manufacturer of the electrodes. 

Electroporation devices were separated into two groups, commercially available and prototype electroporation devices. The devices sold on eBay and similar platforms were also grouped under prototype devices. 

Pulses were first divided in five groups based on pulse duration: short (1–499 µs), medium (500 µs–9.9 ms), long (10 ms or longer), nanosecond pulses (ns) (less than 999 ns), or combinations of pulses (COM). During the review, the need arose, and additional groups were added: exponential pulse (EXP-long, EXP-medium, EXP-short, and EXP-COM), RF-modulated square wave pulse (mod), sinusoidal (SIN), and bipolar square wave pulse (short-BP and medium-BP). 

Biological parameters: The type, concentration, size, promoter of NA, encoded transgene, number of cells used for GET, whether cells were in the suspension or attached, cell line, origin and type of cell line, and electroporation medium were collected. 

Types of NA were further divided into plasmid DNA (pDNA), messenger RNA (mRNA), silencing RNA (siRNA) and others which include guide RNA (gRNA), short hairpin RNA (shRNA), oligonucleotides, chromosomal DNA, polymer-DNA complex, MIDGE vectors, minicircles and complementary DNA (cDNA). 

NA concentrations were divided into 5 categories (<10, 10–50, 51–100, 101–500, >500 µg/mL), size was divided into 4 categories (<2, 2–7, 7–20, >20 kb), and promoters were divided into 2 categories (inducible, constitutive). NA with encoded transgenes were classified as reporter, therapeutic, or empty. 

The number of cells used for GET was divided into 3 categories based on the cell density (<10^5^, 10^5^–10^8^, >10^8^ cells/mL) or attached cells. Cell lines were classified as normal, tumor, or primary cells. 

Reporting of results: Data on GET efficiency and cell survival or overall GET efficiency were collected. Data on the time of measurement and methods of GET efficiency determination were also collected. 

For statistical analysis, the standard deviation or error of the results and the sample size were collected where possible. If it was not clear whether the paper reported the standard deviation or standard error of the mean, then it was assumed to be a standard error. If a range of sample sizes was given, the lowest value was extracted. The online program GraphReader.com (Accessed on 2 May 2022, http://www.graphreader.com/) was used to extract results presented in graphical form if not given in tables or text.

### 2.5. Assessment of Risk of Bias

All included papers were assessed for the risk of bias. Since no standard quality assessment tool exists for in vitro papers, we developed these criteria ourselves. Namely, two reviewers independently assessed the risk of bias for the selected papers. The reporting of each of the electrical and biological parameters of interest was investigated. Based on the below described criteria, the reporting was arranged into three categories, namely appropriate, poor, or inappropriate. 

Because of the poor or inappropriate reporting of at least some parameters in most papers for the overall paper risk of bias assessment, only 10 parameters were chosen (bolded in Table 2) for the further evaluation of papers. The parameters were the controls used, description of electrodes, pulses, pulse generators, concentration, size and promoter of NA, number of cells, electroporation medium, and reporting of the results. Papers were noted as appropriate only if all 10 parameters were appropriately described. If between 5 and 9 of the chosen parameters were described appropriately, then the overall paper risk of bias was poor. If four or fewer of the parameters were described appropriately, then the paper was noted as inappropriate. 

### 2.6. Statistical Analysis

The meta-analysis was performed using MetaLab (available at https://github.com/NMikolajewicz/MetaLab, accessed on 1 October 2020), with a free toolbox for meta-analysis developed in MATLAB R2016b [40]. The main outcome selected from the papers was the overall GET efficiency, which represents the percentage of transfected cells based on the number of cells exposed to the electric pulses. If the overall GET efficiency was not reported, it was calculated as the percentage of transfected cells multiplied by the percentage of viable cells divided by 100. The standard deviation and standard error of the overall GET were calculated from the standard deviations of transfected and viable cells and the sample size according to the error propagation rule. 

The papers with an appropriate overall risk of bias assessment and which also reported the standard deviation or error of the results obtained using different experimental electrical and biological parameters and the sample size were included in further analyses. If the paper reported more than one electrical and/or biological parameters, each result was treated as an independent experiment and included in the meta-analysis. The Cochran Q-test of heterogeneity at a significance level of 5% was applied to evaluate statistical heterogeneity of the data. In addition, H2 and I2 heterogeneity statistics were used to quantitatively assess the heterogeneity of the data. A Baujat plot and single-data exclusion analysis were used to identify the data that contributed most to heterogeneity and affected the outcome. Cumulative data exclusion analysis showed that the threshold for homogeneity of the data set was 79%. Because of statistically significant heterogeneity, a random effect model was used to obtain the pooled overall GET efficiency. Inverse variance weighting was used to calculate the weights of each data set, and 95% confidence intervals (CI) were calculated using a t-distribution. 

Moreover, 19 subgroup analyses were performed according to the type of cell line (normal, tumor, primary), electroporation medium (cell culture media, sugar and salt-based media, commercial electroporation medium, balanced salt solutions), pulses (short, medium, long, exponential and combination of short and long, short-BP, ns), promoter (CMV, SV40, others), and size of NA (<4 kb, between 4 kb and 5 kb, >5 kb). The heterogeneity of each subgroup was statistically significant. Therefore, a random effects model was used to obtain the overall GET efficiency of each subgroup. 

## 3. Results

### 3.1. Paper Selection and Characteristics

**Figure 1:**
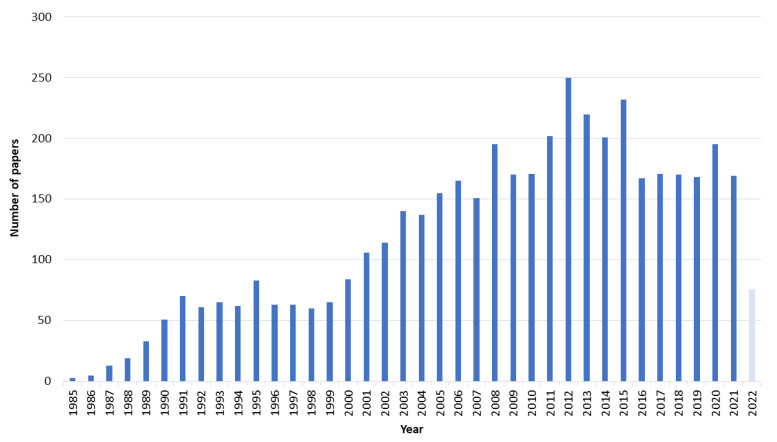
Number of papers retrieved by all 3 searches by year of publication (n = 4525).

All papers were evaluated based on the defined selection criteria, and the risk of bias assessment was performed for appropriate studies. Identifying the year 1982 as the year that this field emerged, we found a remarkable increase in publications concerned with GET up to 2022 (Figure 1). 

**Figure 2:**
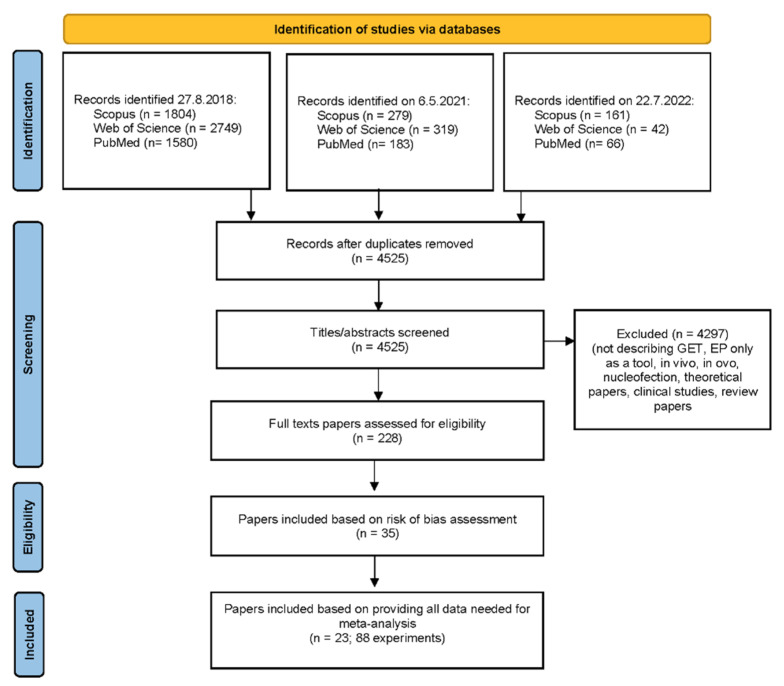
PRISMA flow diagram of paper selection.

The first search in August 2018 retrieved 6133 papers. After the elimination of duplicates and ineligible papers, the full-text versions of the 182 remaining papers were obtained. The second search in May 2021 retrieved an additional 781 papers, of which 32 were eligible for our review. The third search was performed in July 2022. An additional 269 papers were retrieved, of which 14 were eligible to include in our review. Finally, data from 228 eligible papers (Figure 2) were extracted by two investigators [6,13,31,33,37,38,39,41,42,43,44,45,46,47,48,49,50,51,52,53,54,55,56,57,58,59,60,61,62,63,64,65,66,67,68,69,70,71,72,73,74,75,76,77,78,79,80,81,82,83,84,85,86,87,88,89,90,91,92,93,94,95,96,97,98,99,100,101,102,103,104,105,106,107,108,109,110,111,112,113,114,115,116,117,118,119,120,121,122,123,124,125,126,127,128,129,130,131,132,133,134,135,136,137,138,139,140,141,142,143,144,145,146,147,148,149,150,151,152,153,154,155,156,157,158,159,160,161,162,163,164,165,166,167,168,169,170,171,172,173,174,175,176,177,178,179,180,181,182,183,184,185,186,187,188,189,190,191,192,193,194,195,196,197,198,199,200,201,202,203,204,205,206,207,208,209,210,211,212,213,214,215,216,217,218,219,220,221,222,223,224,225,226,227,228,229,230,231,232,233,234,235,236,237,238,239,240,241,242,243,244,245,246,247,248,249,250,251,252,253,254,255,256,257,258,259,260,261] (Supplementary List S1). Of the 228 eligible papers, only 35 papers were appropriate based on the risk of bias assessment [31,37,38,47,48,82,94,105,116,127,130,131,138,139,140,141,144,145,146,157,158,169,173,176,177,186,187,188,189,190,196,199,214,238,253]. For the meta-analysis, data of standard deviation or error and sample size were additionally needed. Of 35 eligible papers based on the risk of bias, standard deviation or error and sample size were only reported in 23 papers [31,37,38,47,48,116,139,140,141,145,146,157,158,169,173,176,177,187,188,190,196,199,253] (Figure 2).

### 3.2. Risk of Bias Assesment

**Figure 3:**
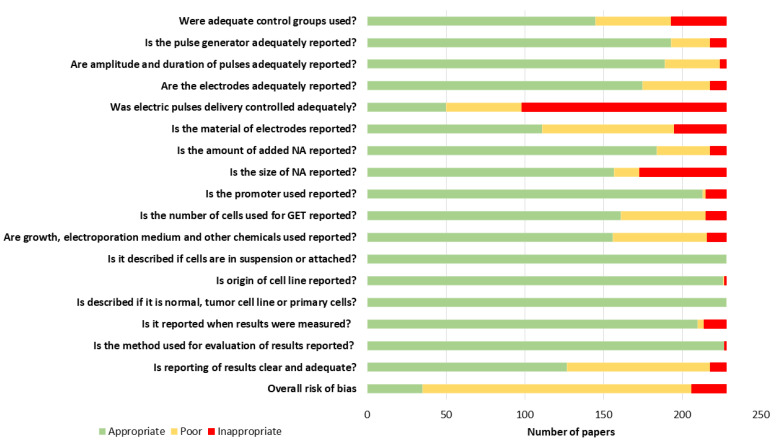
Reporting quality and risk of bias in all 228 papers. Because of the poor or inappropriate reporting of at least some parameters in most papers, only 10 parameters were chosen for the overall paper risk of bias assessment (bolded in Table 2).

The only parameters reported in all papers were whether GET was performed on cells in suspension or attached and the type of cell line, namely tumor, normal, or primary. The method used to measure the results was appropriately reported in all but one paper; similarly, cell origin was appropriately reported in all but two papers. More than 90% of papers appropriately described the promoter of NA and when the results were measured. More than 80% of papers appropriately described the pulse generator, electric pulses, and amount of used NA. Electrodes were appropriately described in 77% of papers, and the number of cells used for GET was appropriately described in 70% of papers. The size of the NA and electroporation medium were appropriately described in 69% of papers. In total, 64% of papers reported the used of adequate control groups. The results were appropriately reported in only 55% of papers, and the material of electrodes was appropriately reported in only 49% of papers. The worst reported parameter was the measurement of output pulses; only 22% of papers appropriately measured delivered pulses (Figure 3). 

### 3.3. Electrodes

Please note that the sum of numbers at each parameter could be higher than the total number of papers used for analysis (228) since one paper could report many experiments using various electrical and/or biological parameters. (For example, a paper describing the use of two different types of electrodes, cuvettes, and wire electrodes, was counted in both categories.)

**Figure 4:**
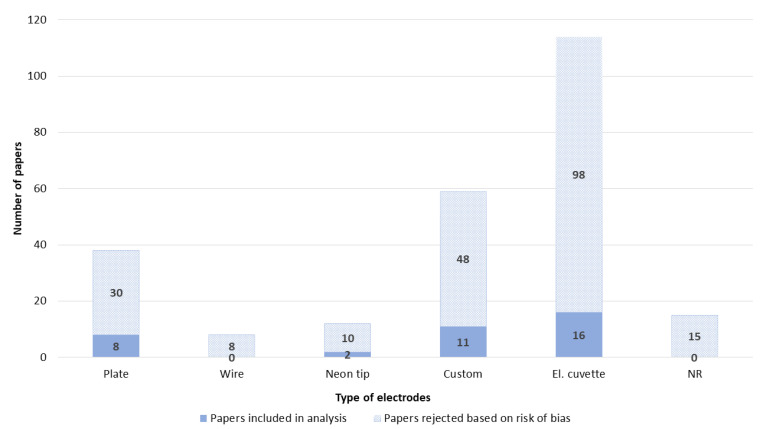
Type of electrodes. NR—not reported.

**Figure 5:**
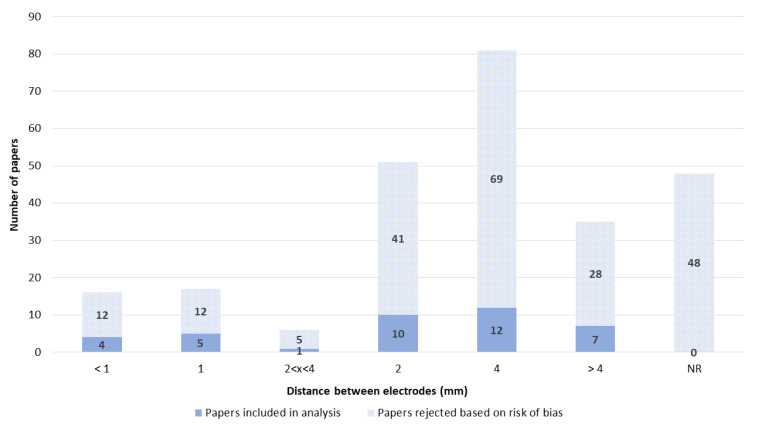
Distance between the electrodes. NR—not reported.

Overall, the electrode type was well reported (Figure 4). By far, the most used were electroporation cuvettes. Therefore, the most common material of electroporation electrodes was aluminum (Supplementary Figure S1), and the distance between the electrodes was 4 mm (Figure 5). The distance between the electrodes, which is critical for electric field calculation, was not well reported. Overall, 48 papers lacked this critical information (Figure 5). 

### 3.4. Electroporation Device

**Figure 6:**
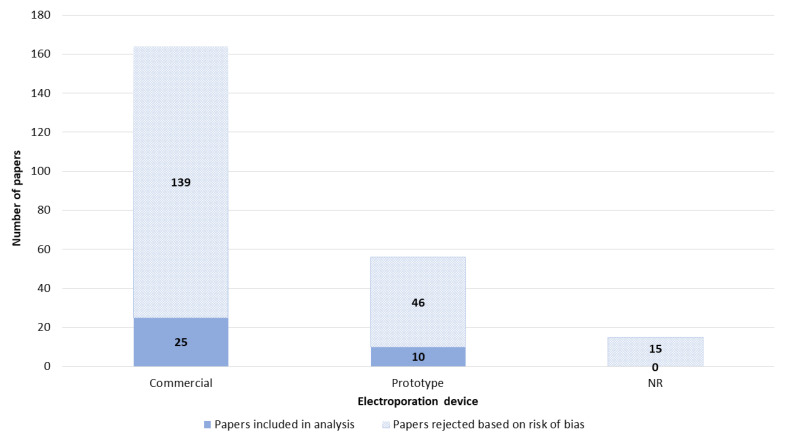
Electroporation device. NR—not reported.

Fifteen papers did not report the data about the electroporation device used. Commercial electroporators were used in the majority of papers. In total, 56 papers reported the use of custom-made, prototype electroporation devices (Figure 6). Among commercial electroporators, the most used were devices produced by Bio-Rad Laboratories, Inc. (Hercules, CA, USA) and BTX^®^ (a division of Harvard Bioscience, Inc., Holliston, MA, USA) (Supplementary Figure S2). 

**Figure 7:**
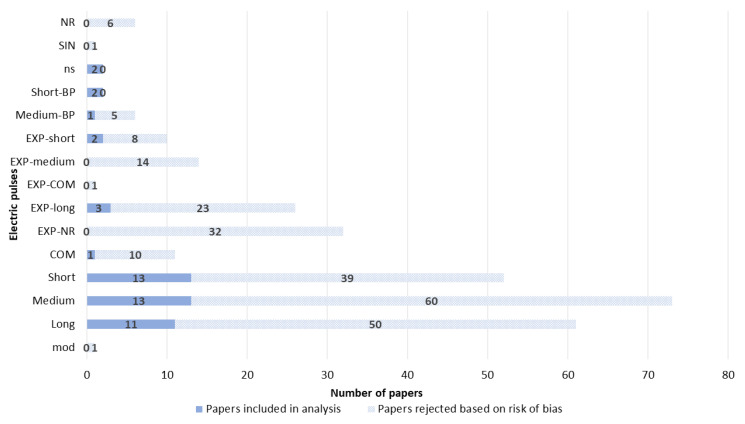
Amplitude and duration of the electric pulses used in the GET experiments. Short: 1–499 µs; medium: 500 µs–9.9 ms; long: 10 ms or longer; nanosecond pulses (ns): less than 999 ns; or combinations of pulses (COM), exponential pulse (EXP-short, EXP-medium, EXP-long, and EXP-COM), RF-modulated square wave pulse (mod), sinusoidal (SIN), bipolar square wave pulse (short-BP and medium-BP), NR—not reported.

Many different types of electrical pulses have been used in GET papers so far. The most use electrical pulse was square wave pulses. Fewer than one-third of papers reported the use of exponential discharge pulses (Figure 7). 

### 3.5. Nucleic Acids

By far, the most used NA was pDNA (>90%). Among RNA molecules, siRNA and mRNA were most often used. Fewer papers reported transfection with oligonucleotides, minicircles, gRNA, and cDNA. Chromosomal DNA, polymer-DNA complex, MIDGE vectors, and shRNA were each used only in one paper (Supplementary Figure S3).

**Figure 8:**
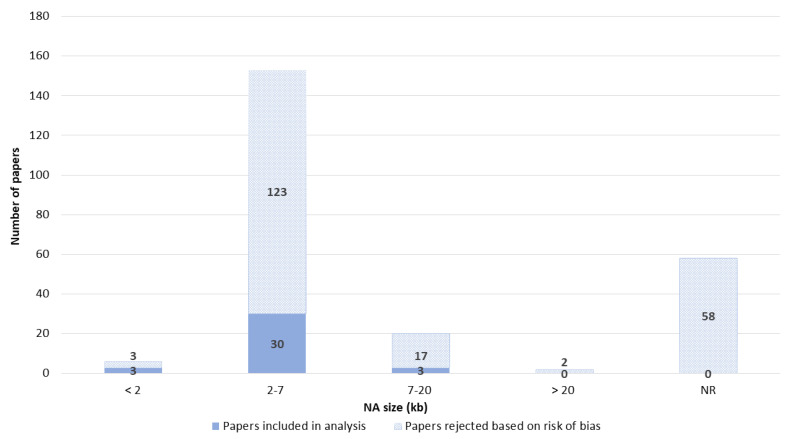
Size of NA. NR—not reported.

The sizes of the NA transfected ranged from 23 bp to 48 kb. Overall, 2–7 kb was the most used size. The size of the NA was one of the categories that was often not reported. Around 25% of papers did not report the NA size (Figure 8). 

**Figure 9:**
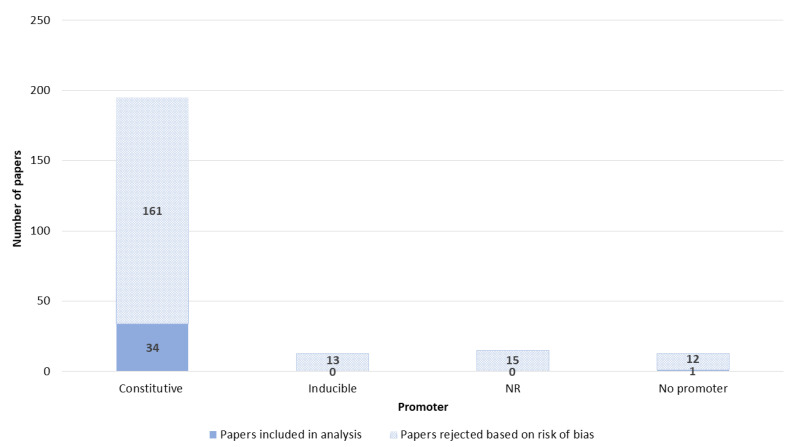
Promoter of NA. NR—not reported.

The promoters were divided into constitutive and inducible promoters. The majority of papers reported the use of constitutive promoters (Figure 9). Among constitutive promoters, CMV and SV40 were used most often. 

The majority of transgenes in the papers included in this study were reporters (>90%). In eight papers, NAs that did not encode the transgene were used. They were classified as empty (Supplementary Figure S4).

**Figure 10:**
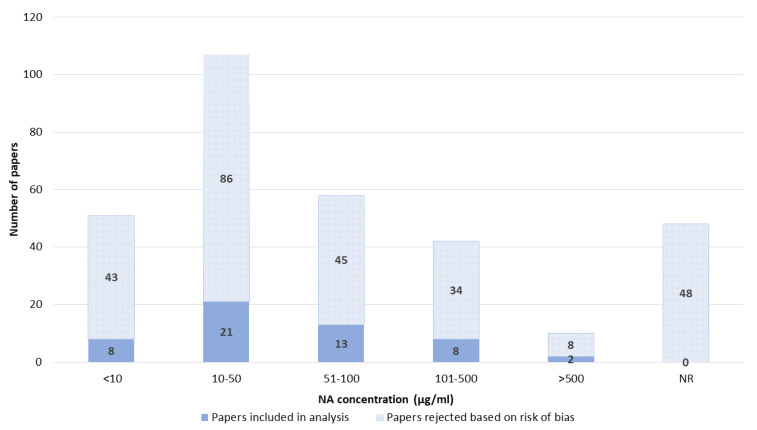
NA concentration. NR—not reported.

The NA concentration was also often not reported. Most papers reported concentrations between 10 and 50 µg/mL. Concentrations lower than 10 µg/mL and higher concentrations of NA were also reported; in particular, 51–100 µg/mL and 101–500 µg/mL were also commonly used. Concentrations higher than 500 µg/mL were rarely reported (Figure 10). 

### 3.6. Cell Lines

**Figure 11:**
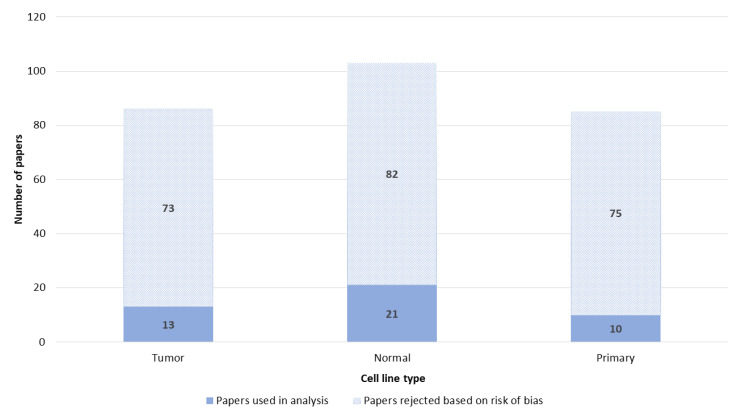
Type of cell line.

Cell lines were divided into three groups, namely tumor, normal or primary cells. All three groups of cells were used in a similar number of papers (Figure 11).

The cells originated from 14 different species, with human, mouse, and hamster being the most often used, followed by rat and monkey. Other origins that were less common were goat, rabbit, dog, swine, cat, chicken, buck, bovine, and sheep. In two papers, the origin of the cell line was not reported (Supplementary Figure S5). 

Cells were more frequently electroporated in the suspension compared to the electroporation of attached cells (Supplementary Figure S6).

**Figure 12:**
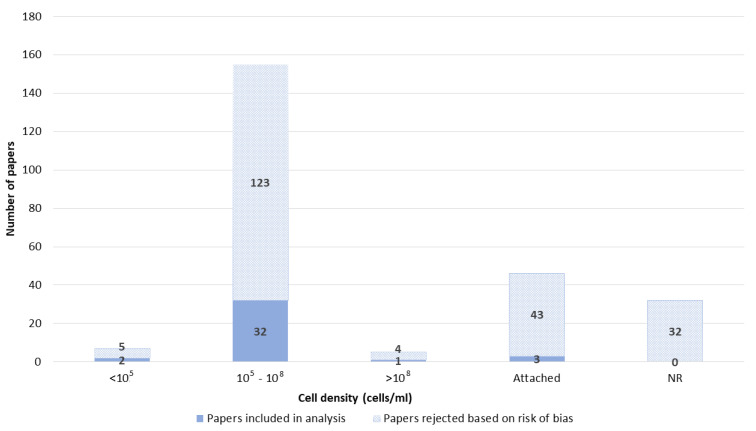
Cell density. NR–not reported.

The density of cells electroporated in the suspension was divided into three groups: up to 10^5^, 10^5^–10^8^, and higher than 10^8^ cells per milliliter. Most papers reported GET with a cell density between 10^5^ and 10^8^ (Figure 12).

### 3.7. Electroporation Medium

**Figure 13:**
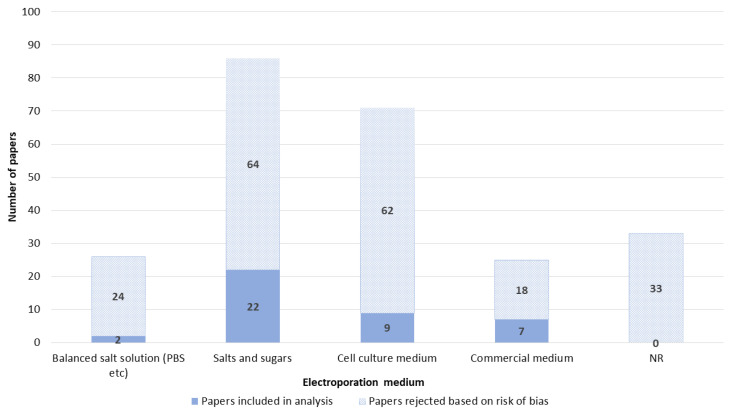
Electroporation medium. NR—not reported.

The media used for GET were divided into four groups. Most often, GET was performed in media consisting of salts and sugars, followed by GET in cell culture medium, GET in balanced salt solutions, and GET in commercial electroporation medium. The GET medium was not reported in 33 papers (Figure 13). 

### 3.8. Time at Which GET Was Measured

**Figure 14:**
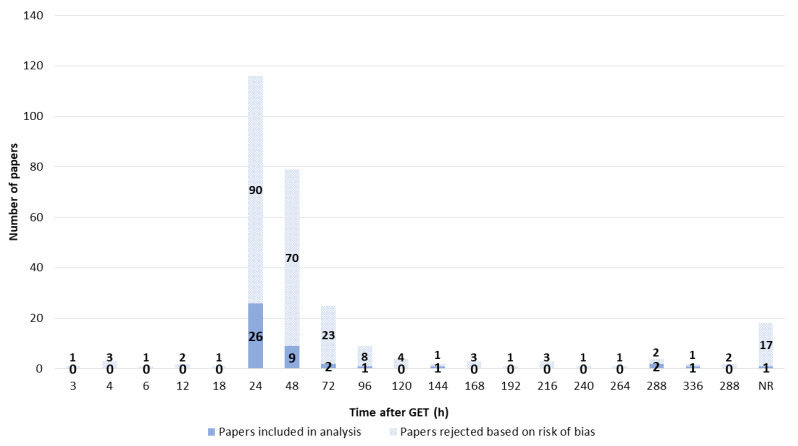
Time at which GET was measured.

The time at which GET was measured ranged from 3 to 288 h (12 days) after GET. GET was most often measured 24 or 48 h after GET, followed by 72 h after GET. Overall, 18 papers did not report when GET was measured (Figure 14). 

### 3.9. Sets of Parameters That Result in the Most Efficient GET

The highest overall GET efficiency (86.49%) using pDNA under the control of the constitutive CMV promoter was reported by Zhou et al. in 2020. Adherent HeLa cells in a sugar- and salt-containing electroporation buffer were exposed to an overall equal electric field intensity (100 V/cm) for 0.5 ms by individually addressable sequentially energizing microelectrodes arranged in a nested and non-contact manner [157]. 

A high overall GET efficiency (80.75%) was also obtained by Cegovnik and Novaković in 2003. They exposed a suspension of B16F1 cells in a sugar- and salt-containing electroporation buffer with the addition of pDNA under the control of the constitutive SV40 promoter to exponentially decreasing and electronically controlled electric pulses in an electroporation cuvette [173].

A somewhat lower but still high overall GET efficiency (77.26%) was also published by Zu et al. in 2020. They constructed a micropillar array of electrodes integrated as the top and bottom wall in a horizontal flow chamber. Then, 293T cells were exposed to an electric field of 625 × 10^4^ V/cm in a cell culture medium for 10 ms in the presence of pDNA under the control of the constitutive CMV promoter [146].

### 3.10. Pooled Overall GET Efficiency

All 10 parameters for the overall paper risk of bias assessment were appropriately described in 35 papers, but only 23 papers also reported the standard deviation or error of the results and the sample size. Therefore, only these 23 papers were included in the analysis, which, together, reported 143 overall GET efficiencies obtained using different experimental electrical and biological parameters and could therefore be treated as independent experiments (Supplementary Table S1). Since electric pulses were divided only by pulse length and the electric field was not a separate electrical parameter, we included 88 optimal GET efficiencies disregarding the electric field.

**Figure 15:**
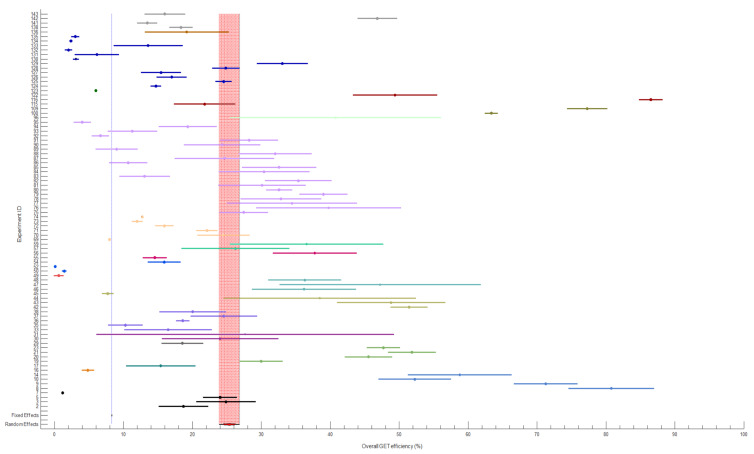
Forest plot showing the mean of each experiment (circle) with 95% CI (line) along with the estimate of the global overall GET efficiency (red band) using the random effect model. The size of the circle reflects the weight of the experiment in the pooling. The experiments reported in the same paper are colored with the same color. The experimental parameters of each experiment marked with experimental IDs can be found in Supplementary Table S1.

The meta-analysis using the random effect model revealed that the pooled overall GET efficiency was 25% (95% CI, 23.8–26.9) (Figure 15). According to the results of the I^2^ test, there was a high degree of heterogeneity in the primary meta-analysis (I^2^ = 99.94%, *p* < 0.001).

### 3.11. Subgroup Analyses

Subgroup analysis was conducted according to the type of cell line (normal, tumor, primary), electroporation medium (cell culture media, sugar and salt-based media, commercial electroporation media, balanced salt solutions), pulses (short, medium, long, exponential and combination of short and long, short-BP, ns), promoter (CMV, SV40, others), and size of NA (<4 kb, between 4 kb and 5 kb, >5 kb). The results of the subgroup analyses are given in Table 3.

From the cell line point of view, the overall GET efficiency in the tumor cell lines (39.9%; 95% CI: ±17.3%) is statistically significantly higher than the overall GET efficiency in the primary cell lines (15.2%; 95% CI: ±2.18%) and normal cell lines (23.6%; 95% CI: ±1.8%). In addition, the overall GET efficiency in the normal cell lines is statistically significantly higher than the overall GET efficiency in the primary cell lines. In terms of electroporation medium, the two media with the highest overall GET efficiency were balanced salt solutions (33.4%; 95% CI, ±20.9%) and cell culture media (30.1%; 95% CI, ±9.0%). However, none of them were statistically significantly higher than the overall GET efficiency, which was obtained after GET in sugar and salt-based media and in commercial electroporation media. For electrical pulses, the overall GET efficiency after GET with exponential and short-long pulses (50.6%; 95% CI: ±12.9%) is statistically significantly higher than the overall GET efficiencies after GET with other types of pulses, while the overall GET efficiencies according to GET with other types of pulses are not statistically different. In terms of the promoter, the overall GET efficiency after GET with the promoter SV40 (51.2%; 95% CI, ±29.3%) is statistically significantly higher than the overall GET efficiency after GET with the promoter CMV (23.8%; 95% CI, ±1.6%) and the overall GET efficiency after GET with other promoters (7.6%; 95% CI, ±4.1%). In addition, the overall GET efficiency after GET with the promoter CMV is statistically significantly higher than overall GET efficiency after GET with other promoters. From the perspective of NA size, the subgroup analysis shows that the overall GET efficiencies according to GET with different sizes of NA are not statistically different. The set of continuous NA size variables was additionally evaluated in a univariable meta-regression analysis. The results showed that the size of the NA was not correlated with the overall efficiency of GET (slope = −1.19, R^2^ = 0.0094, *p* = 0.37). Using meta-regression analysis, we also checked the dependence of the overall GET efficiency on the concentration of the NA. The results demonstrated that concentration of NA was not correlated with the overall GET efficiency (R^2^ = 0.00485, *p* = 0.52).

## 4. Discussion

The interest in the use of GET has steadily increased since it was first reported in 1982. Our systematic literature search revealed approximately 4600 papers reporting the use of GET in various research studies and biotechnological applications. In this study, we focused on the main experimental electrical and biological parameters affecting the efficiency of GET under in vitro conditions. A large number of electrical and biological parameters affect the efficiency of GET, which made GET protocol optimization more complex. It also led to a high number of different GET protocols used in different papers, making it difficult to identify the optimal one(s). Using a systematic review, we collected the papers reporting the efficiency of GET as a function of the change in at least one experimental electrical or biological parameter. In the meta-analysis, the overall GET efficiency was evaluated, and the influence of the electrical and biological parameters that affected the overall GET efficiency most were studied. 

The experimental electrical parameters included the type and geometry of electrodes, the material of the electrodes, the device used to apply the electrical pulses (type and manufacturer), the amplitude and duration of the electrical pulses, and the method used to measure the output pulses. The experimental biological parameters included the type, dosage, size, promoter of NA, encoded transgene, number of cells used for EP, whether the cells were electrotransfected in the suspension or attached, cell line, the origin and type of cell line, and the electroporation medium. To allow a fair and unbiased evaluation of the GET experimental result, the electrical and biological parameters must be specified, appropriate controls must be included in the experiment, and the efficiency of GET with the method of evaluation must be indicated. Although in 228 papers, the efficiency of GET as a function of the change in at least one electrical or biological parameter was reported, only 23 papers contained all desired information and were therefore reliable enough to include in the meta-analysis. All experiments reported in 23 papers resulted in a pooled overall GET efficiency of 25% (95% CI, 23.8–26.9). The highest overall GET efficiency was above 85% [157]. The combinations of parameters that led to high overall GET differed greatly, so we cannot recommend specific protocol as the most efficient. From this, we can see that the optimization of GET protocol for each set of parameters is needed. 

Due to the broad field of GET in this study, we focused only on the in vitro reports that describe the use of different electrical and/or biological parameters. The papers that reported only the electroporation program used but did not specify details of delivered electric pulses, such as duration, number, and amplitude, could not be included in our study. Therefore, for example, the papers using only “nucleofection” were excluded.

The risk of bias assessment was performed on 228 papers and included 17 parameters which were previously reported to influence GET efficiency. After the risk of bias assessment, we realized that some parameters were appropriately reported in only a few papers. Among the electric parameters, the measurement of output pulses and electrode material were the least reported. Biological parameters were reported more frequently than electrical parameters; for example, the type of cell line and whether GET was performed on the cell suspension or on the attached cells were reported in all papers. 

For the meta-analysis, we reduced the number of parameters and the number of categories describing each parameter to the type of cell line, electroporation medium, electric pulses, promoter, and size of the NA. Although each parameter was initially divided into several categories in order to describe all the different variants used in the experiments, some categories were not represented in the experiments included in the meta-analysis, so we merged similar categories.

Control groups are an essential part of any scientific experiment, yet 15% of papers included in our review did not report the use of appropriate controls. GET experiments include exposing cells to NAs and electric pulses, and the effect of each should be monitored with appropriate controls. Therefore, GET experiments should include three controls: cells exposed only to electric pulses, cells exposed only to NA, and cells exposed to neither NA nor electric pulses.

The review of the collected papers revealed that, although the type of electrodes used in the experiments was generally specified, more than 20% of the papers lacked geometric parameters of the electrodes (e.g., the distance between parallel plate electrodes) that would allow the determination of the electric field. Although it is known that high-voltage electric pulses cause electrode oxidation and metal corrosion, which change the electrical properties of the electrodes and the chemical composition of the surrounding medium depending on the electrode material [18], the material of the electrodes was not specified in 15% of the papers. Electroporation cuvettes were the most reported type of electrodes and are a good choice because they are industrially manufactured with an accurate distance between the electrodes. However, in the last few years, low-cost cuvettes of questionable quality have also become available on the market. Therefore, researchers should always also report the manufacturer of used electroporation cuvettes. 

In the case of appropriate device use and development, custom-made, prototype electroporation devices can sometimes be even more reliable than commercially available devices. In 85% of the papers, the authors stated the electroporation device used in the experiments, but only 22% of the papers mentioned the measurement or evaluation of the output pulses. For successful and efficient GET, it is necessary that the output pulses are well defined, and only the measurement of the pulses in each experiment ensures that the pulses are delivered as desired. In most studies, rectangular monopolar electric pulses were used, which replaced exponentially decaying pulses that have been used in the early years. 

The meta-analysis showed that the overall GET efficiency after GET with exponential and short-long pulses is statistically significantly higher than the overall GET efficiencies after GET with other types of pulses. These results are expected and confirm the importance of both cell membrane permeabilization and electrophoretic component of electric pulses in GET in vitro [11].

Among biological parameters, only the type of cell line and whether GET was performed on the suspension or attached cells were described in all 228 papers. The method used for the evaluation of GET was reported in all but one paper, and the origin of the cell line was reported in all but two papers. It was previously shown that GET efficiency depends on the cell line [11]. Therefore, it is important that the cell line origin and type are described.

Further, more than 90% of papers appropriately reported when results were measured and the NA promoter. The plasmid must contain the necessary elements for expression in mammalian cells. A proper promoter should be used to express the transgene. The transgene can be expressed constitutively (it transcribes constantly) or inducible (its transcription is controlled) [262]. Among constitutive promoters, CMV and SV40 were used most often, which is in agreement with the literature reports that the viral promoters CMV, RSV, and SV40 are the most widely used [262]. Although CMV was most often used promoter in papers included in our systematic review, the meta-analysis showed that the SV40 promoter led to a significantly higher overall GET.

In addition, the time at which GET was measured is important since different NA have different expression profiles. GET was most often measured after 24–72 h, with more than 40% of papers reporting measuring of GET after 24 h. We recently showed that the time dynamics of expression of the same NA can vary between cell lines [11]. Based on this, it is important to report on when GET was measured and attempt different times of measurement to detect the peak of expression. 

The NA concentration and number of cells used for GET were appropriately reported in 80% and 70% of papers, respectively. NA concentration is one of the parameters which is reported to influence GET. A higher NA concentration usually leads to a higher GET [11,37,38]. Contrary to expectations, our meta-analysis did not show a significant effect of NA concentration on the overall GET. Previously, it was shown that the effect of a higher NA concentration depends on the applied pulses. The increase of GET with the increasing NA concentration was more pronounced when short (micro or nanosecond) pulses were used, while with longer pulses, a plateau in GET efficiency with increasing NA concentration was observed [11,37]. All pulse types were included in the meta-analysis. Since shorter pulses, even with higher NA concentration, led to comparable (or lower) GET efficiency compared to longer pulses, this could explain why NA concentration was not a significant parameter in our meta-analysis.

Cell suspension density can influence the process of cell membrane permeabilization. In a dense suspension (>10^8^ cells/mL), pulses with higher amplitudes are needed to achieve cell membrane permeabilization compared to more dilute cell suspension. In addition, cell swelling, which occurs after cell membrane permeabilization, can limit the accessibility and transport of NA between the cells and to the membrane [263]. Further, the number of cells in the suspension also determines the distance between the cell and NA, with higher cell density resulting in a smaller distance between the cells and NA [11]. Both cell membrane permeabilization and the distance between the cell and NA influence GET efficiency, meaning that the number of cells used for GET should always be reported. 

The biological parameters which were most poorly reported were size of NA and electroporation medium. Both categories were adequately described only in 69% of papers. Contrary to our meta-analysis, some papers showed that NA size and electroporation medium can affect GET. NA of smaller size was reported to enable higher GET [32,264,265]. Our meta-analysis did not show a significant effect of NA size on the overall GET. Papers which were included in meta-analysis mostly reported the use of two commercially available reporter pDNA-encoding green fluorescent proteins with sizes 3.5 and 4.7 kb. The difference in size between the pDNA was small and may have been too small to have an effect on GET. Similar results were observed in a previous study [11]. Poor reporting of NA size might also show that authors do not think NA size is important. This might be because mobility and distribution of NA in the cell suspension is higher compared to the in vivo distribution in tissue where NA size represents a bigger obstacle [2]. 

Interestingly, our meta-analysis showed the highest overall GET efficiency when balanced salt solutions and cell culture media were used. Although the difference was not statistically significant, we would still expect higher overall GET efficiency in commercial electroporation media, which were supposedly optimized. 

Conductivity and composition of electroporation medium can affect GET directly by affecting the cell membrane permeabilization or formation of NA aggregates on the cell membrane, or indirectly by affecting cell survival [3,33,141,266]. Consequently, the medium used for GET should always be reported. In addition, the electroporation medium composition also contributes to chemical and temperature changes during pulse delivery depending on the pulse type and amplitude [37,267].

There are different ways of GET efficiency measurement, most often depending on GET application. Nevertheless, to determine the overall GET and be able to compare the GET efficiency between different papers, data on cell survival are also needed. In total, 75% of papers in our review only reported transfection efficiency without also providing data on cell survival. Only 15% of papers reported both transfection efficiency and cell survival. 

Based on the abovementioned poor reporting of electrical and biological parameters affecting GET, we prepared a table of recommendations for reporting (Table 4).

## 5. Conclusions

For now, there is no single or universal GET strategy that is appropriate for all cell lines and aims. Apart from the availability of the required electroporation device and electrodes, the choice of an optimal GET approach or strategy depends on factors such as the electroporation medium; type and origin of cells; and the size, concentration, promoter, and type of the nucleic acids to be transfected. Equally important are the inclusion of appropriate controls in the GET experiment and the measurement or evaluation of the output pulses to allow a fair and unbiased evaluation of the experimental results. Our systematic literature review of different electrical and biological parameters effect on in vitro GET efficiency showed a pooled overall GET efficiency of 25%. The parameters which significantly affected overall GET efficiency were the type of cell line, nucleic acids promoter, and pulse parameters. Overall, GET was significantly higher in tumor cell lines compared to normal and primary cell lines, and in normal cell lines compared to primary cell lines. Overall, GET was also significantly higher when the SV40 promoter was used compared to CMV or other promoters. In addition, exponential and short-long pulses led to significantly higher overall GET compared to all other pulses. However, it needs to be highlighted that these results were obtained based on the meta-analysis of only 23 papers out of 228. This low number of analyzed papers was the result of an incredible and unacceptable lack of reporting of essential information in GET in vitro papers. Since many experimental electrical and biological parameters can affect GET efficiency, it is important that all used parameters are adequately reported to enable the comparison of results, as well as potentially faster and more efficient experiment planning and optimization in further studies. Only the adequate and detailed reporting of all used parameters can enable successful future meta-analyses that could elucidate which electrical and/or biological parameters significantly contribute to higher GET efficiency. 

## Figures and Tables

**Table 1:** Summary of all abbreviations used throughout the paper.

cDNA	Complementary DNA
CI	Confidence intervals
CMV	Cytomegalovirus
COM	Combination of pulses
DNA	Deoxyribonucleic acid
EXP-COM	Combination of exponential pulses
EXP-long	Long exponential pulses
EXP-medium	Medium exponential pulses
EXP-short	Short exponential pulses
HF-BP	High-frequency bipolar pulses
GET	Gene electrotransfer
gRNA	Guide RNA
medium-BP	Medium bipolar square wave pulse
MIDGE	Minimalistic, immunologically defined gene expression
mod	RF modulated square wave pulse
mRNA	Messenger RNA
NA	Nucleic acid
ns	Nanosecond pulses
pDNA	Plasmid DNA
RF	Radio frequency
RNA	Ribonucleic acid
short-BP	Short bipolar square wave pulse
shRNA	Short hairpin RNA
SIN	Sinusoidal pulses
siRNA	Silencing RNA
SV40	Simian virus 40

**Table 2:** Criteria for assessment of risk of bias.

	Appropriate	Poor	Inappropriate
Controls			
**Were adequate control groups used?**	At least control group without electroporation	Just control group without pDNA; not clear if it is control group without electroporation or without pDNA; other control groups	Not reported
**Electrical parameters**			
**Is the pulse generator adequately reported?**	Output pulse parameters or design of the electroporator is known	The type of the electroporator is reported but the specifications of the output pulses or the design is not reported	Not reported
**Are amplitude and duration of pulses adequately reported?**	Amplitude and duration of pulses is reported	Amplitude and pulse waveform is known, but not the time constant	Not reported
**Are the electrodes adequately reported?**	Distance between the electrodes and the design (geometry to determine electric field distribution) is reported	Distance between the electrodes is reported but not the design (geometry to determine electric field distribution)	Not reported
Was electric pulses delivery controlled adequately?	The user measures output pulses with an oscilloscope	The user measures or in any other way assesses output pulses; pulse generators that measure output pulses	Not reported
Is the material of electrodes described?	Material of solid electrodes is reported or thickness and material of basal and plated material of the electrodes is reported	Basal and plated materials are reported but not the thickness	Not reported
**Biological parameters**			
**Is the amount of added NA reported?**	Amount or concentration and volume	Just concentration or range reported	Not reported
**Is the size of NA reported?**	Size is described or manufacturer is reported	Not reported for all used pDNA-s	Not reported
**Is the promoter used reported?**	Promoter is reported or manufacturer is reported	Not reported for all used pDNA-s	Not reported
**Is the number of cells used for GET reported?**	Number is reported	Just concentration or range reported	Not reported
**Are growth, electroporation medium, and other chemicals used reported?**	Growth medium, electroporation medium and other chemicals used are reported	Electroporation medium is not reported	Not reported
Is it described if cells are in suspension or attached?	Described if cells are in suspension or attached	/	Not reported
Is the origin of cell line reported?	Origin of cell line is reported	Origin is not reported for all cell lines	Not reported
Is it described if it is normal, tumor cell line or primary cells?	Described if it is normal, tumor cell line or primary cells	/	Not reported
Is it reported when results were measured?	Reported when GET was measured	Just time frame reported	Not reported
Is the method used for evaluation of results reported?	Method used for evaluation of GET efficiency is reported	/	Not reported
**Is reporting of results clear and adequate? (Defined if it is percentage of GET, overall GET)**	Percentage of transfection and survival are reported	Only percentage of transfection is reported	Not reported

**Table 3:** Results of the subgroup analysis. According to the results of the I^2^ test, there was a high degree of heterogeneity in all subgroups (I^2^ > 98%).

Parameter	No. of Experiments (No. of Papers)	Overall GET Efficiency (%) (95% CI)
Normal	53 (15)	23.6 (±1.8)
Tumor	16 (7)	39.9 (±17.3)
Primary	19 (7)	15.2 (±2.18)
Electroporation medium		
Sugar and salt-based media	43 (16)	22.8 (±2.0)
Cell culture media	32 (6)	30.1 (±9.0)
Commercial electroporation media	10 (4)	20.1 (±6.9)
Balanced salt solutions	3 (2)	33.4 (±20.9)
Pulses		
Short	25 (9)	17.6 (± 1.9)
Medium	19 (10)	28.2 (± 12.4)
Long	20 (7)	28.2 (±4.4)
Short-long and exp	9 (3)	50.6 (±12.9)
BP	8 (2)	16.2 (±9.9)
ns	7 (2)	15.7 (±11.6)
Promoter		
CMV	76 (20)	23.8 (±1.6)
SV40	8 (2)	51.2 (±29.3)
others	4 (2)	7.6 (±4.1)
Size of NA		
<4 kb	19 (9)	26.2 (±2.6)
between 4 kb and 5 kb	51 (11)	25.0 (±4.2)
>5 kb	18 (5)	24.1 (±2.7)

**Table 4:** Recommendations for reporting of parameters of in vitro GET experiments.

Parameter	What Should Be Included/Reported
**Controls**	Cells which were not exposed to EP and NA Cells with NA but not exposed to EP Cells without NA but exposed to EP
**Pulse Generator**	Manufacturer and type In the case of prototype devices, the construction design should be reported. *
**Electric pulses**	Shape, duration, number, pulse repetition frequency and amplitude. Pulse delivery should always be monitored by an appropriate device and at least two figures of delivered pulses should be provided, one of a single pulse zoomed and another with reduced time scale, where all delivered pulses are displayed. *
**Electrodes**	Manufacturer and type of electrodes In the case of custom-made electrodes geometry, material should be reported, and in case of microelectrodes, the construction procedure should also be reported. *
**NA concentration**	Final NA concentration or amount of added NA and volume of suspension
**NA size**	Manufacturer and catalog number or specified NA size
**Promoter**	Manufacturer and catalog number or specified promoter
**Number of cells**	Number of cells exposed to GET or cell concentration and volume of suspension
**Electroporation medium**	Manufacturer and catalog number or specified composition of electroporation medium
**Cells**	Manufacturer and catalog number or specified type and origin of cell line. It should also be reported if cells were treated in suspension or attached.
**Results**	Transfection efficiency and cell survival should be reported. In addition, the method and time of transfection efficiency and cell survival measurement should be described.

* see also [268,269].

## Data Availability

Data are available from the corresponding author on request.

## Supplementary Materials

The following supporting information can be downloaded at: https://www.mdpi.com/article/10.3390/pharmaceutics14122700/s1, Figure S1: Material of the electrodes; Figure S2: Commercial electroporation device manufacturers; Figure S3: Type of nucleic acid; Figure S4: Transgene coded by nucleic acid; Figure S5: Origin of cell line; Figure S6: Electroporation of cells in suspension or attached cells; List S1: A list of all 228 papers used in systematic review; Table S1: Overall GET efficiencies obtained using different experimental electrical and biological parameters.
