# Supplementary material for: Effect of Experimental Electrical and Biological Parameters on Gene Transfer by Electroporation: A Systematic Review and Meta-Analysis
Source: Pharmaceutics. 2022 Dec 2;14(12):2700. doi: 10.3390/pharmaceutics14122700 (PMC9786189; doi:10.3390/pharmaceutics14122700)
Supplement: Supplementary file 1 [file pharmaceutics-14-02700-s001.zip › Supplementary figure.pdf]

Supplementary information for

"Effect of experimental electrical and biological parameters on gene transfer by electroporation – a review"

Tjaša Potočnik [1], Alenka Maček Lebar [1], Špela Kos [2], Matej Reberšek [1], Eva Pirc [1], Gregor Serša [2] and Damijan Miklavčič [1,*]

[1] University of Ljubljana, Faculty of Electrical Engineering, Tržaška 25, 1000 Ljubljana, Slovenia
[2] Institute of Oncology Ljubljana, Zaloška cesta 2, 1000 Ljubljana, Slovenia
* Correspondence: damijan.miklavcic@fe.uni-lj.si

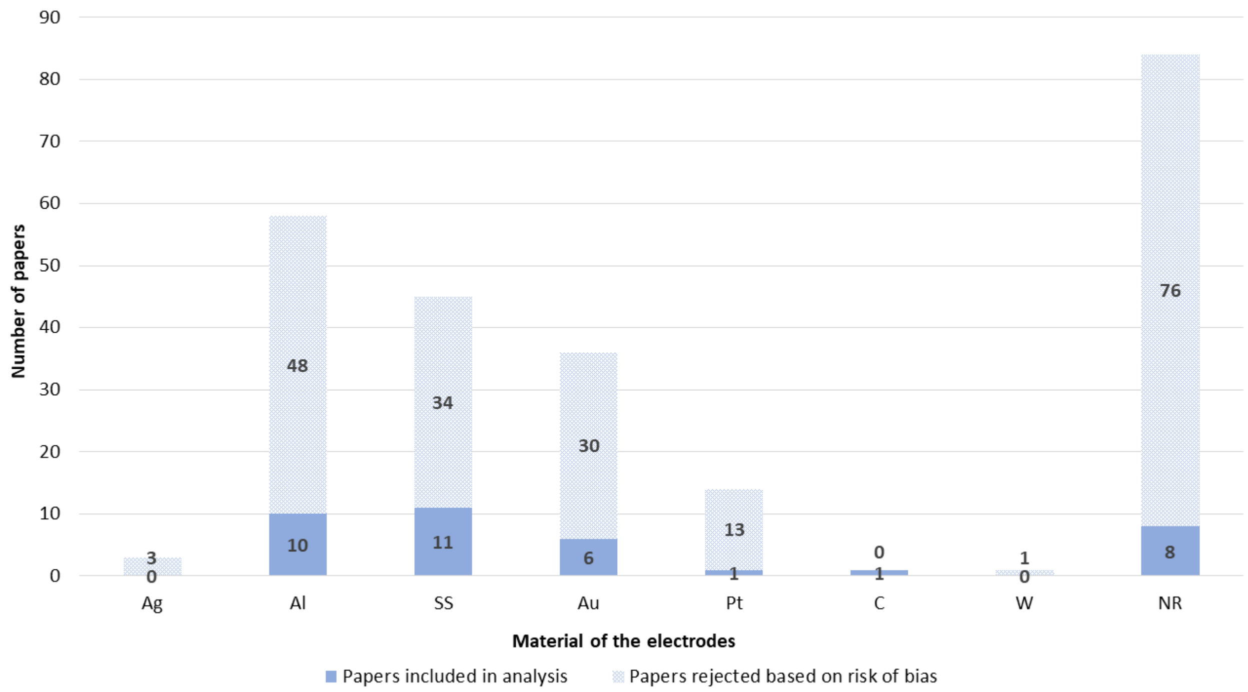

Supplementary Figure S1: Material of the electrodes. Silver (Ag), Aluminum (Al), Stainless Steel (SS), Gold (Au), Platinum (Pt), Carbon (C), Wolfram (W), NR – not reported.

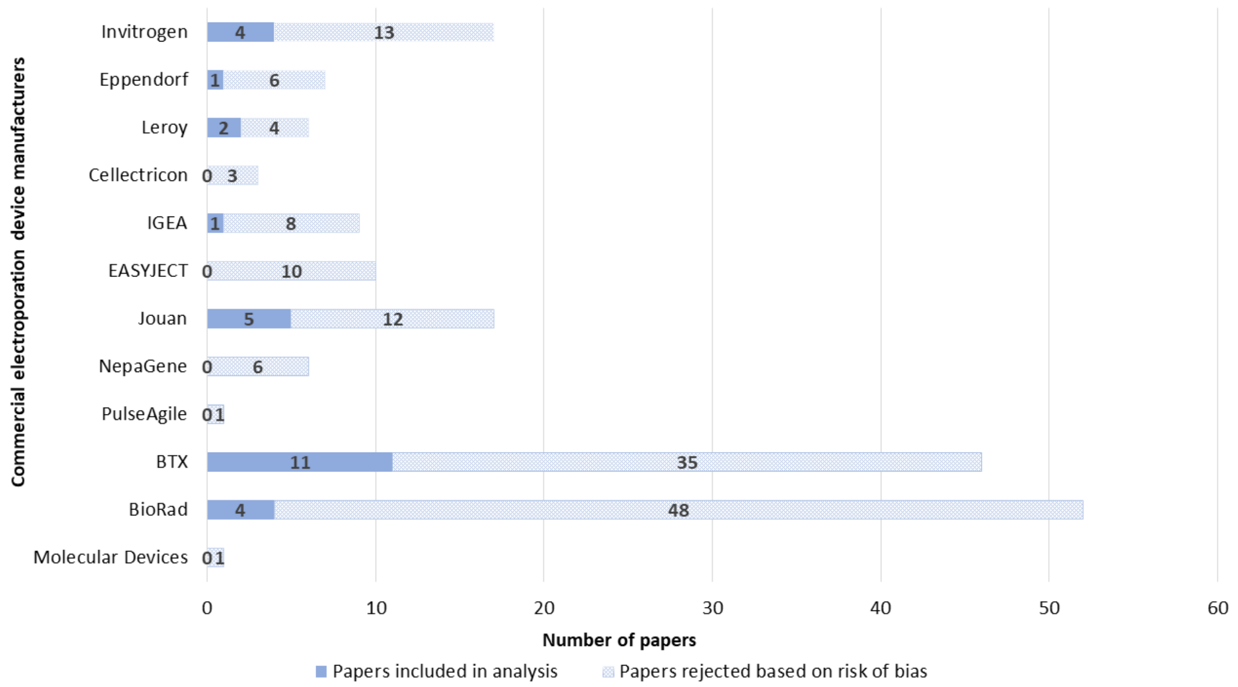

Supplementary Figure S2: Commercial electroporation device manufacturers.

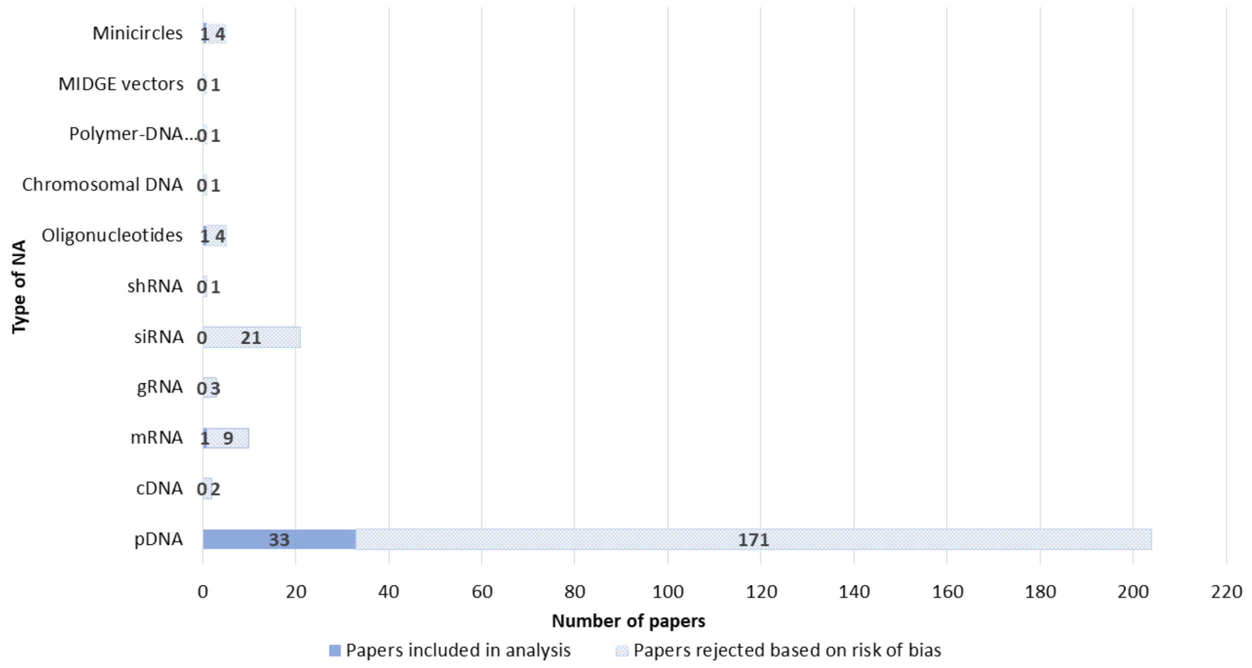

Supplementary Figure S3: Type of nucleic acid.

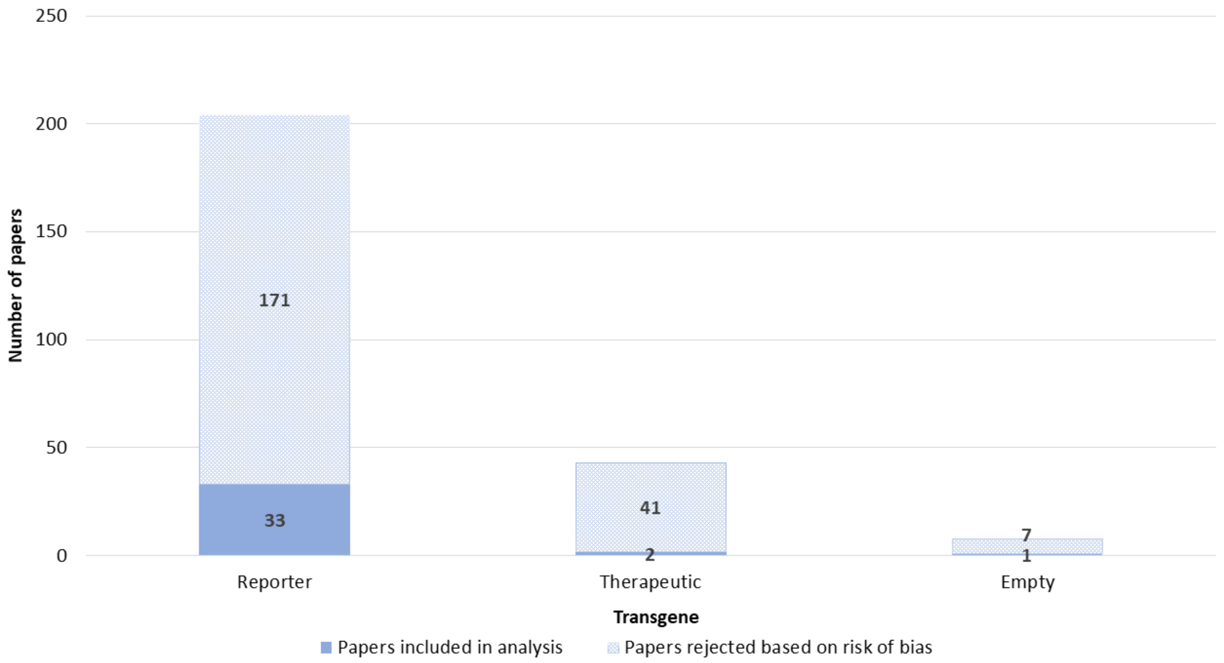

Supplementary Figure S4: Transgene coded by nucleic acid.

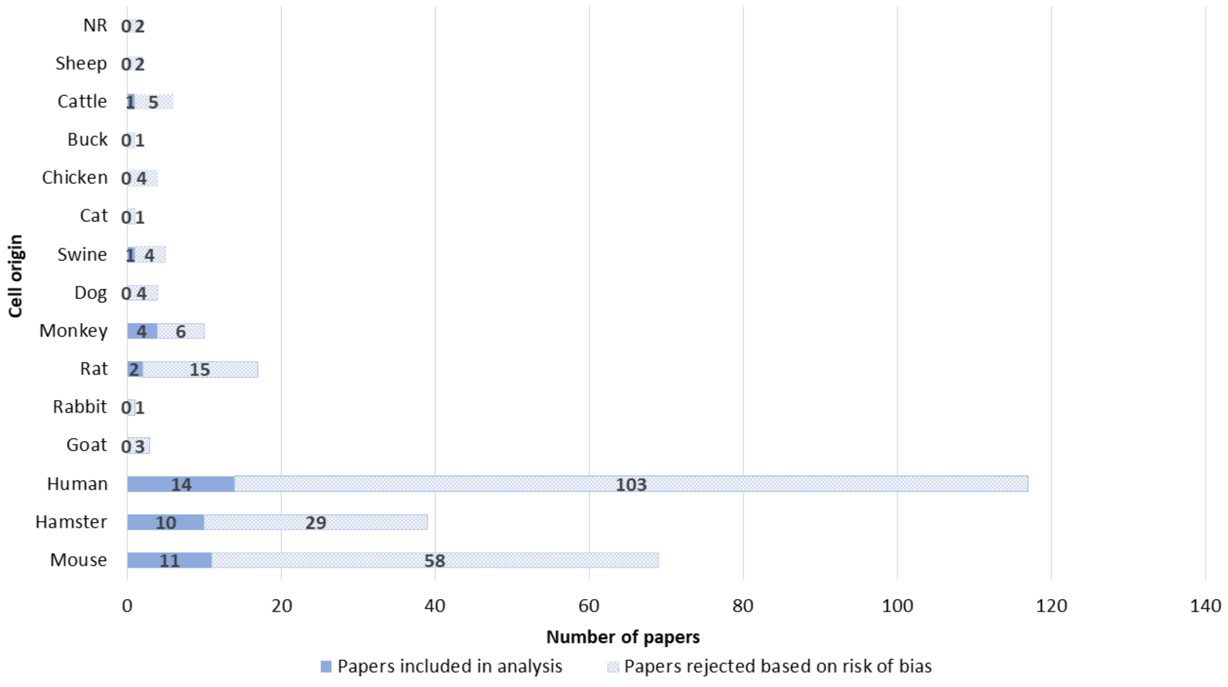

Supplementary Figure S5: Origin of cell line. NR – not reported.

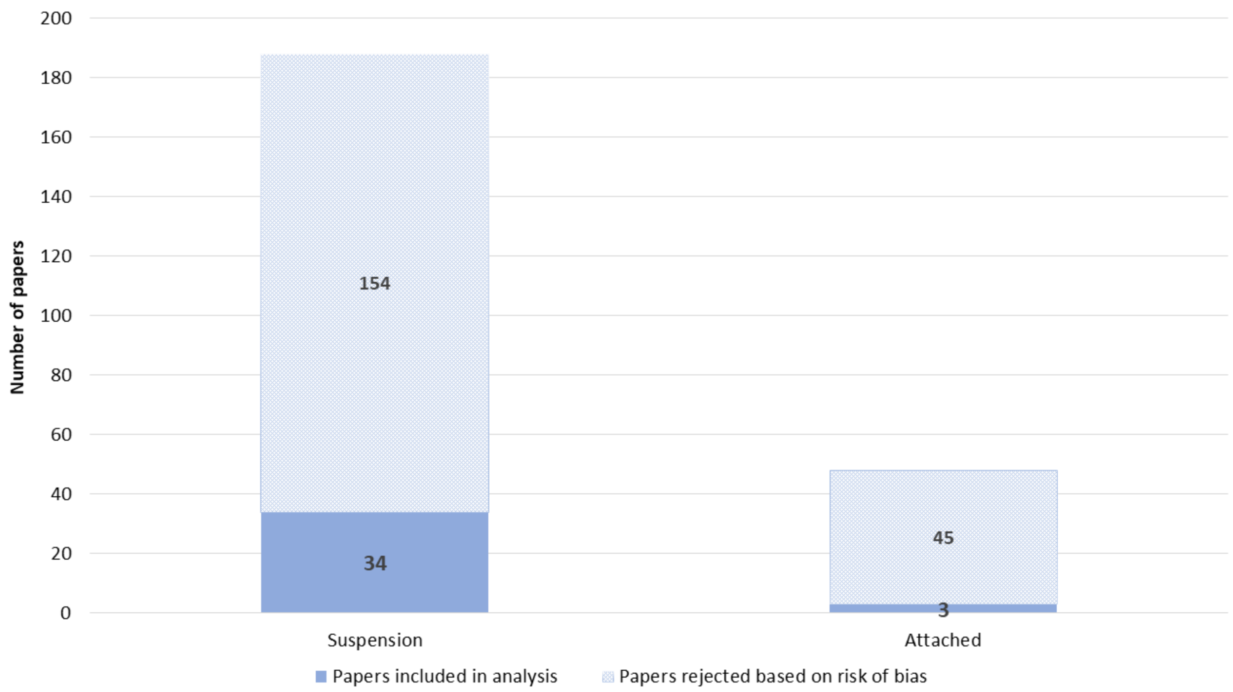

Supplementary Figure S6: Electroporation of cells in suspension or attached cells.

Supplementary List S1: A list of all 288 papers used in systematic review.

1. Abdul Halim, N.S.S.; Fakiruddin, K.S.; Ali, S.A.; Yahaya, B.H.; Halim, N.; Fakiruddin, K.S.; Ali, S.A.; Yahaya, B.H. A Comparative Study of Non-Viral Gene Delivery Techniques to Human Adipose-Derived Mesenchymal Stem Cell. **2014**, *15*, doi:10.3390/ijms150915044.

2. Amiri Yekta, A.; Dalman, A.; Sanati, M.H.; Fatemi, N.; Vazirinasab, H.; Zomorodipour, A.; Chehrazi, M.; Gourabi, H. Optimization of The Electroporation Conditions for Transfection of Human Factor IX into The Goat Fetal Fibroblasts. *Cell J.* **2013**, *14*, 270–275.

3. Anderson, M.L.M.M.L.M.; Spandidos, D.A.D.A.; Coggins, J.R.J.R. Electroporation of Lymphoid Cells: Factors Affecting the Efficiency of Transfection. *J. Biochem. Biophys. Methods* **1991**, *22*, 207–222, doi:10.1016/0165-022X(91)90069-9.

4. Andreason, G.L.; Evans, G.A. Optimization of Electroporation for Transfection of Mammalian Cell Lines. **1989**, *180*, doi:10.1016/0003-2697(89)90429-6.

5. Bodwell, J.; Swift, F.; Richardson, J. Long Duration Electroporation for Achieving High Level Expression of Glucocorticoid Receptors in Mammalian Cell Lines. *J. Steroid Biochem. Mol. Biol.* **1999**, *68*, 77–82, doi:10.1016/S0960-0760(98)00162-9.

6. Bosnjak, M.; Jesenko, T.; Kamensek, U.; Sersa, G.; Lavrencak, J.; Heller, L.; Cemazar, M. Electrotransfer of Different Control Plasmids Elicits Different Antitumor Effectiveness in B16.F10 Melanoma. *Cancers (Basel).* **2018**, *10*, 37, doi:10.3390/cancers10020037.

7. Bosnjak, M.; Lorente, B.C.; Pogacar, Z.; Makovsek, V.; Cemazar, M. Different Incubation Times of Cells After Gene Electrotransfer in Fetal Bovine Serum Affect Cell Viability, but Not Transfection

Efficiency. **2014**, *247*, doi:10.1007/s00232-014-9649-9.

8. Bradshaw, H.D.; Parson, W.W.; Sheffer, M.; Lioubin, P.J.; Mulvihill, E.R.; Gordon, M.P. Design, Construction, and Use of an Electroporator for Plant Protoplasts and Animal Cells. *Anal. Biochem.* **1987**, *166*, 342–348.

9. Bravo, S.B.; Garcia-Rendueles, M.E.R.R.; Perez-Romero, S.; Cameselle-Teijeiro, J.; Rodrigues, J.S.; Barreiro, F.; Alvarez, C. V Expression of Exogenous Proteins and Short Hairpin RNAs in Human Primary Thyrocytes. **2010**, *400*, doi:10.1016/j.ab.2010.01.034.

10. Browne, C.J.; Pinyon, J.L.; Housley, D.M.; Crawford, E.N.; Lovell, N.H.; Klugmann, M.; Housley, G.D. Mapping of Bionic Array Electric Field Focusing in Plasmid DNA-Based Gene Electrotransfer. *Gene Ther.* **2016**, *23*, 369–379, doi:10.1038/gt.2016.8.

11. Buchser, W.J.W.J.; Pardinas, J.R.J.R.; Shi, Y.; Bixby, J.L.J.L.J.L.; Lemmon, V.P.V.P. 96-Well Electroporation Method for Transfection of Mammalian Central Neurons. *Biotechniques* **2006**, *41*, doi:10.2144/000112279.

12. Bulysheva, A.A.A.; Burcus, N.; Lundberg, C.; Edelblute, C.M.C.M.; Francis, M.P.M.P.; Heller, R. Recellularized Human Dermis for Testing Gene Electrotransfer Ex Vivo. **2016**, *11*, 35002, doi:10.1088/1748-6041/11/3/035002.

13. Bureau, M.F.F.; Gehl, J.; Deleuze, V.; Mir, L.M.M.; Scherman, D. Importance of Association between Permeabilization and Electrophoretic Forces for Intramuscular DNA Electrotransfer. **2000**, *1474*, doi:10.1016/S0304-4165(00)00028-3.

14. Burrin, J.M.; Jameson, J.L. Regulation of Transfected Glycoprotein Hormone Alpha-Gene Expression in Primary Pituitary Cell Cultures. *Mol. Endocrinol.* **1989**, *3*, 1643–1651, doi:10.1210/mend-3-10-1643.

15. Cao, F.; Xie, X.; Gollan, T.; Zhao, L.; Narsinh, K.; Lee, R.J.; Wu, J.C. Comparison of Gene-Transfer Efficiency in Human Embryonic Stem Cells. **2010**, *12*, 15–24, doi:10.1007/s11307-009-0236-x.

16. Cegovnik, U. Setting Optimal Parameters for in Vitro Electrotransfection of B16F1, SA1, LPB, SCK, L929 and CHO Cells Using Predefined Exponentially Decaying Electric Pulses. *Bioelectrochemistry* **2003**, doi:10.1016/j.biolechem.2003.10.009.

17. Cemazar, M.; Sersa, G.; Wilson, J.; Tozer, G.M.G.M.; Hart, S.L.S.L.; Grosel, A.; Dachs, G.U. Effective Gene Transfer to Solid Tumors Using Different Nonviral Gene Delivery Techniques: Electroporation, Liposomes, and Integrin-Targeted Vector. **2002**, *9*, 399–406, doi:10.1038/sj.cgt.7700454.

18. Cepurniene, K.; Ruzgys, P.; Treinys, R.; Satkauskiene, I.; Satkauskas, S.; Čepurnienė, K.; Ruzgys, P.; Treinys, R.; Šatkauskienė, I.; Šatkauskas, S.; et al. Influence of Plasmid Concentration on DNA Electrotransfer in Vitro Using High-Voltage and Low-Voltage Pulses. **2010**, *236*, doi:10.1007/s00232-010-9270-5.

19. Cervia, L.D.; Chang, C.-C.; Wang, L.; Mao, M.; Yuan, F. Enhancing Electrotransfection Efficiency through Improvement in Nuclear Entry of Plasmid DNA. **2018**, *11*, 263–271, doi:10.1016/j.omtn.2018.02.009.

20. Cesnulevicius, K.; Timmer, M.; Wesemann, M.; Thomas, T.; Barkhausen, T.; Grothe, C. Nucleofection Is the Most Efficient Nonviral Transfection Method for Neuronal Stem Cells

Derived from Ventral Mesencephali with No Changes in Cell Composition or Dopaminergic Fate. *Stem Cells* **2006**, *24*, 2776–2791, doi:10.1634/stemcells.2006-0176.

21. Chabot, S.; Orio, J.; Castanier, R.; Bellard, E.; Nielsen, S.J.; Golzio, M.; Teissié, J. LNA-Based Oligonucleotide Electrotransfer for MiRNA Inhibition. *Mol. Ther.* **2012**, *20*, 1590–1598, doi:10.1038/mt.2012.95.

22. Chabot, S.; Orio, J.; Schmeer, M.; Schleef, M.; Golzio, M.; Teissié, J. Minicircle DNA Electrotransfer for Efficient Tissue-Targeted Gene Delivery. **2013**, *20*, doi:10.1038/gt.2011.215.

23. Chopinet, L.; Wasungu, L.; Rols, M.P. First Explanations for Differences in Electrotransfection Efficiency in Vitro and in Vivo Using Spheroid Model. In Proceedings of the International Journal of Pharmaceutics; 2012.

24. Coulberson, A.L.; Hud, N. V; LeDoux, J.M.; Vilfan, I.D.; Prausnitz, M.R. Gene Packaging with Lipids, Peptides and Viruses Inhibits Transfection by Electroporation in Vitro. *J. Control. Release* **2003**, *86*, 361–370, doi:10.1016/S0168-3659(02)00417-0.

25. Delgado-Cañedo, A.; dos Santos, D.G.; Chies, J.A.B.; Kvitko, K.; Nardi, N.B. Optimization of an Electroporation Protocol Using the K562 Cell Line as a Model: Role of Cell Cycle Phase and Cytoplasmic DNAses. *Cytotechnology* **2006**, *51*, doi:10.1007/s10616-006-9028-1.

26. Delteil, C.; Teissié, J.; Rols, M.-P. Effect of Serum on in Vitro Electrically Mediated Gene Delivery and Expression in Mammalian Cells. *Biochim. Biophys. Acta - Biomembr.* **2000**, *1467*, 362–368, doi:10.1016/S0005-2736(00)00235-2.

27. Donate, A.; Heller, R. Assessment of Delivery Parameters with the Multi-Electrode Array for Development of a DNA Vaccine against Bacillus Anthracis. *Bioelectrochemistry* **2013**, *94*, 1–6, doi:10.1016/j.bioelechem.2013.04.004.

28. Dullaers, M.; Breckpot, K.; Van Meirvenne, S.; Bonehill, A.; Tuyaerts, S.; Michiels, A.; Straetman, L.; Heirman, C.; De Greef, C.; Van Der Bruggen, P.; et al. Side-by-Side Comparison of Lentivirally Transduced and MRNA-Electroporated Dendritic Cells: Implications for Cancer Immunotherapy Protocols. **2004**, *10*, doi:10.1016/j.ymthe.2004.07.017.

29. Ear, T.; Giguère, P.; Fleury, A.; Stankova, J.; Payet, M.D.; Dupuis, G. High Efficiency Transient Transfection of Genes in Human Umbilical Vein Endothelial Cells by Electroporation. *J. Immunol. Methods* **2001**, *257*, 41–49.

30. Escoffre, J.-M.; Nikolova, B.; Mallet, L.; Henri, J.; Favard, C.; Golzio, M.; Teissié, J.; Tsoneva, I.; Rols, M.-P. New Insights in the Gene Electrotransfer Process: Evidence for the Involvement of the Plasmid DNA Topology. *Curr. Gene Ther.* **2012**, *12*, 417–422, doi:10.2174/156652312802762554.

31. Escoffre, J.M.M.; Kaddur, K.; Rols, M.P.P.; Bouakaz, A. In Vitro Gene Transfer by Electrosonoporation. **2010**, *36*, doi:10.1016/j.ultrasmedbio.2010.06.019.

32. Eslaminejad, T.; Nematollahi-Mahani, S.N.; Ansari, M. Cationic $β$-Cyclodextrin-Chitosan Conjugates as Potential Carrier for PmCherry-C1 Gene Delivery. *Mol. Biotechnol.* **2016**, *58*, 287–298, doi:10.1007/s12033-016-9927-0.

33. Espinos, E.; Liu, J.H.; Bader, C.R.; Bernheim, L. Efficient Non-Viral DNA-Mediated Gene Transfer to Human Primary Myoblasts Using Electroporation. *Neuromuscul. Disord.* **2001**, *11*, 341–349, doi:10.1016/S0960-8966(00)00204-2.

34. Faurie, C.; Rebersek, M.; Golzio, M.; Kanduser, M.; Escoffre, J.-M.; Pavlin, M.; Teissie, J.; Miklavcic, D.; Rols, M.-P. Electro-Mediated Gene Transfer and Expression Are Controlled by the Life-Time of DNA/Membrane Complex Formation. *J. Gene Med.* **2010**, *12*, 117–125, doi:10.1002/jgm.1414.

35. Faurie, C.; Phez, E.; Golzio, M.; Vossen, C.; Lesbordes, J.-C.J.-C.; Delteil, C.; Teissié, J.; Rols, M.-P.M.-P. Effect of Electric Field Vectoriality on Electrically Mediated Gene Delivery in Mammalian Cells. **2004**, *1665*, 92–100, doi:10.1016/j.bbamem.2004.06.018.

36. Feril, L.B.; Ogawa, R.; Tachibana, K.; Kondo, T. Optimized Ultrasound-Mediated Gene Transfection in Cancer Cells. **2006**, *97*, doi:10.1111/j.1349-7006.2006.00286.x.

37. Ferreira, E.; Potier, E.; Logeart-Avramoglou, D.; Salomskaite-Davalgiene, S.; Mir, L.M.M.; Petite, H. Optimization of a Gene Electrotransfer Method for Mesenchymal Stem Cell Transfection. **2008**, *15*, doi:10.1038/gt.2008.9.

38. Fountain, J.W.; Lockwood, W.K.; Collins, F.S. Transfection of Primary Human Skin Fibroblasts by Electroporation. *Gene* **1988**, *68*, 167–172, doi:10.1016/0378-1119(88)90610-5.

39. García-Sánchez, T.; Guitart, M.; Rosell-Ferrer, J.; Gómez-Foix, A.M.; Bragós, R. A New Spiral Microelectrode Assembly for Electroporation and Impedance Measurements of Adherent Cell Monolayers. *Biomed. Microdevices* **2014**, *16*, 575–590, doi:10.1007/s10544-014-9860-6.

40. Ghartey-Tagoe, E.B.; Babbin, B.A.; Nusrat, A.; Neish, A.S.; Prausnitz, M.R. Plasmid DNA and SiRNA Transfection of Intestinal Epithelial Monolayers by Electroporation. **2006**, *315*, doi:10.1016/j.ijpharm.2006.02.022.

41. Ghosh, C.; Song, W.; Lahiri, D.K. Efficient DNA Transfection in Neuronal and Astrocytic Cell Lines. *Mol. Biol. Rep.* **2000**, *27*, 113–121, doi:10.1023/A:1007173906990.

42. Giroux, S.J.D.D.; Alves-Leiva, C.; Lécluse, Y.; Martin, P.; Albagli, O.; Godin, I. Gene Transfer to Pre-Hematopoietic and Committed Hematopoietic Precursors in the Early Mouse Yolk Sac: A Comparative Study between in Situ Electroporation and Retroviral Transduction. **2007**, *7*, 79, doi:10.1186/1471-213X-7-79.

43. Goldstein, S.; Fordis, C.M.; Howard, B.H. Enhanced Transfection Efficiency and Improved Cell Survival after Electroporation of G2/M-Synchronized Cells and Treatment with Sodium Butyrate. *Nucleic Acids Res.* **1989**, *17*, 3959–3971, doi:10.1093/nar/17.10.3959.

44. Golzio, M.; Teissié, J.; Rols, M.P. Cell Synchronization Effect on Mammalian Cell Permeabilization and Gene Delivery by Electric Field. *Biochim. Biophys. Acta - Biomembr.* **2002**, *1563*, 23–28, doi:10.1016/S0005-2736(02)00369-3.

45. Grabarek, J.B.; Plusa, B.; Glover, D.M.; Zernicka-Goetz, M. Efficient Delivery of DsRNA into Zona-Enclosed Mouse Oocytes and Preimplantation Embryos by Electroporation. *genesis* **2002**, *32*, 269–276, doi:10.1002/gene.10076.

46. Grund, E.M.; Muise-Helmericks, R.C. Cost Efficient and Effective Gene Transfer into the Human Natural Killer Cell Line, NK92. **2005**, *296*, doi:10.1016/j.jim.2004.10.008.

47. Guo, H.; Hao, R.; Wei, Y.; Sun, D.; Sun, S.; Zhang, Z. Optimization of Electrotransfection Conditions of Mammalian Cells with Different Biological Features. **2012**, *245*, doi:10.1007/s00232-012-9480-0.

48. Haberl, S.S.; Pavlin, M. Use of Collagen Gel as a Three-Dimensional In Vitro Model to Study Electropermeabilization and Gene Electrotransfer. **2010**, *236*, 87–95, doi:10.1007/s00232-010-9280-3.

49. Harrison, R.L.; Byrne, B.J.; Tung, L. Electroporation-Mediated Gene Transfer in Cardiac Tissue. *FEBS Lett.* **1998**, *435*, 1–5, doi:10.1016/S0014-5793(98)00987-9.

50. Hashemi, A.; Roohvand, F.; Ghahremani, M.H.; Aghasadeghi, M.R.; Vahabpour, R.; Motevali, F.; Memarnejadian, A. Optimization of Transfection Methods for Huh-7 and Vero Cells: A Comparative Study. *Cytol. Genet.* **2012**, *46*, 347–353, doi:10.3103/S0095452712060035.

51. Helledie, T.; Nurcombe, V.; Cool, S.M. A Simple and Reliable Electroporation Method for Human Bone Marrow Mesenchymal Stem Cells. **2008**, *17*, doi:10.1089/scd.2007.0209.

52. Hernández, J.L.; Coll, T.; Ciudad, C.J. A Highly Efficient Electroporation Method for the Transfection of Endothelial Cells. *Angiogenesis* **2004**, *7*, 235–241, doi:10.1007/s10456-004-4180-8.

53. Hilliard, C.M.; Fletcher, S.; Yeoh, G.C.T. Calcium Phosphate Transfection and Cell-Specific Expression of Heterologous Genes in Primary Fetal Rat Hepatocytes. **1996**, *28*, doi:10.1016/1357-2725(96)00007-6.

54. Hoerauf, W.W.W.; Cazares, V.A.V.A.V.A.; Subramani, A.; Stuenkel, E.L.E.L. Efficient Transfection of Dissociated Mouse Chromaffin Cells Using Small-Volume Electroporation. *Cytotechnology* **2015**, *67*, doi:10.1007/s10616-014-9699-y.

55. Hornstein, B.D.B.D.B.D.; Roman, D.; Arévalo-Soliz, L.M.L.M.; Engevik, M.A.M.A.; Zechiedrich, L. Effects of Circular DNA Length on Transfection Efficiency by Electroporation into HeLa Cells. **2016**, *11*, e0167537, doi:10.1371/journal.pone.0167537.

56. Huang, H.; Wei, Z.; Huang, Y.; Zhao, D.; Zheng, L.; Cai, T.; Wu, M.; Wang, W.; Ding, X.; Zhou, Z.; et al. An Efficient and High-Throughput Electroporation Microchip Applicable for SiRNA Delivery. *Lab Chip* **2011**, *11*, 163–172, doi:10.1039/c0lc00195c.

57. Huang, K.-S.; Lin, Y.-C.; Su, C.-C.; Fang, C.-S. Enhancement of an Electroporation System for Gene Delivery Using Electrophoresis with a Planar Electrode. *Lab Chip* **2007**, *7*, 86–92, doi:10.1039/B613753A.

58. Huang, S.; Deshmukh, H.; Rajagopalan, K.K.; Wang, S. Gold Nanoparticles Electroporation Enhanced Polyplex Delivery to Mammalian Cells. **2014**, *35*, doi:10.1002/elps.201300617.

59. Hui, S.W.; Stoicheva, N.; Zhao, Y.L. High-Efficiency Loading, Transfection, and Fusion of Cells by Electroporation in Two-Phase Polymer Systems. *Biophys. J.* **1996**, *71*, 1123–1130, doi:10.1016/S0006-3495(96)79314-1.

60. Ishibashi, T.; Takoh, K.; Kaji, H.; Abe, T.; Nishizawa, M. A Porous Membrane-Based Culture Substrate for Localized in Situ Electroporation of Adherent Mammalian Cells. *Sensors Actuators B Chem.* **2007**, *128*, 5–11, doi:10.1016/j.snb.2007.05.027.

61. Iversen, N.; Birkenes, B.; Torsdalen, K.; Djurovic, S. Electroporation by Nucleofector Is the Best Nonviral Transfection Technique in Human Endothelial and Smooth Muscle Cells. *Genet. Vaccines Ther.* **2005**, *3*, 2, doi:10.1186/1479-0556-3-2.

62. Jain, T.; Muthuswamy, J. Bio-Chip for Spatially Controlled Transfection of Nucleic Acid Payloads into Cells in a Culture. **2007**, *7*, doi:10.1039/b707479d.

63. Jiang, C.-K.; Connolly, D.; Blumenberg, M. Comparison of Methods for Transfection of Human Epidermal Keratinocytes. *J. Invest. Dermatol.* **1991**, *97*, 969–973, doi:10.1111/1523-1747.ep12491889.

64. Joergensen, M.; Agerholm-Larsen, B.; Nielsen, P.E.; Gehl, J. Efficiency of Cellular Delivery of Antisense Peptide Nucleic Acid by Electroporation Depends on Charge and Electroporation Geometry. *Oligonucleotides* **2011**, *21*, 29–37, doi:10.1089/oli.2010.0266.

65. Jordan, E.T.E.T.; Collins, M.; Terefe, J.; Ugozzoli, L.; Rubio, T. Optimizing Electroporation Conditions in Primary and Other Difficult-to-Transfect Cells. **2008**, *19*.

66. Kalina, J.; Kolmanová, A.; Mikuš, T.; Mičáková, A.; Trefil, P. Transfection of Cock Spermatogonial Cells via Electroporation and Lipofection. *Czech J. Anim. Sci.* **2003**, *48*, 279–284.

67. Kang, J.-H.J.-H.; Toita, R.; Niidome, T.; Katayama, Y. Effective Delivery of DNA into Tumor Cells and Tissues by Electroporation of Polymer–DNA Complex. **2008**, *265*, doi:10.1016/j.canlet.2008.02.045.

68. Kawabata, I.; Umeda, T.; Yamamoto, K.; Okabe, S. Electroporation-Mediated Gene Transfer System Applied to Cultured CNS Neurons. *Neuroreport* **2004**, *15*, 971–975.

69. Kim, J.A.; Lee, W.G.; Jung, N.C. Enhanced Electro-Mediated Gene Delivery Using Carrier Genes. **2010**, *78*, doi:10.1016/j.bioelechem.2009.08.012.

70. Kim, J.A.J.A.; Cho, K.; Shin, Y.S.Y.S.; Jung, N.; Chung, C.; Chang, J.K.J.K. A Multi-Channel Electroporation Microchip for Gene Transfection in Mammalian Cells. **2007**, *22*, doi:10.1016/j.bios.2007.02.009.

71. Kim, J.A.J.A.; Lee, W.G.W.G. Role of Weakly Polarized Nanoparticles in Electroporation. *Nanoscale* **2011**, *3*, doi:10.1039/c0nr00448k.

72. Klenchin, V.A.; Sukharev, S.I.; Serov, S.M.; Chernomordik, L. V; Chizmadzhev YuA Electrically Induced DNA Uptake by Cells Is a Fast Process Involving DNA Electrophoresis. *Biophys. J.* **1991**, *60*, 804–811, doi:10.1016/S0006-3495(91)82115-4.

73. Knutson, J.C.; Yee, D. Electroporation: Parameters Affecting Transfer of DNA into Mammalian Cells. *Anal. Biochem.* **1987**, *164*, 44–52, doi:10.1016/0003-2697(87)90365-4.

74. Kobelt, D.; Schleef, M.; Schmeer, M.; Aumann, J.; Schlag, P.M.; Walther, W. Performance of High Quality Minicircle DNA for In Vitro and In Vivo Gene Transfer. **2013**, *53*, doi:10.1007/s12033-012-9535-6.

75. Kos, S.; Tesic, N.; Kamensek, U.; Blagus, T.; Cemazar, M.; Kranjc, S.; Lavrencak, J.; Sersa, G. Improved Specificity of Gene Electrotransfer to Skin Using PDNA Under the Control of Collagen Tissue-Specific Promoter. *J. Membr. Biol.* **2015**, *248*, 919–928, doi:10.1007/S00232-015-9799-4.

76. Kotnis, R.A.A.; Thompson, M.M.M.; Eady, S.L.L.; Budd, J.S.S.; Bell, P.R.F.R.; James, R.F.L.F. Optimisation of Gene Transfer into Vascular Endothelial Cells Using Electroporation. **1995**, *9*, doi:10.1016/S1078-5884(05)80228-X.

77. Kumar Pramod, R.; Kumar, R.; Mitra, A. Transgenic Expression of Green Fluorescent Protein in

Caprine Embryos Produced through Electroporation-Aided Sperm-Mediated Gene Transfer. *Gene* **2016**, *576*, doi:10.1016/j.gene.2015.10.066.

78. Kwee, S.; Nielsen, H. V; Celis, J.E. Electropermeabilization of Human Cultured Cells Grown in Monolayers. Incorporation of Monoclonal Antibodies. **1990**, 65–80.

79. Lakshmipathy, U.; Pelacho, B.; Sudo, K.; Linehan, J.L.; Coucouvanis, E.; Kaufman, D.S.; Verfaillie, C.M. Efficient Transfection of Embryonic and Adult Stem Cells. **2004**, *22*, doi:10.1634/stemcells.22-4-531.

80. Lam, A.P.; Dean, D.A. Cyclic Stretch-Induced Nuclear Localization of Transcription Factors Results in Increased Nuclear Targeting of Plasmids in Alveolar Epithelial Cells. **2008**, *10*, doi:10.1002/jgm.1187.

81. Lampreht Tratar, U.; Kos, S.; Kamensek, U.; Ota, M.; Tozon, N.; Sersa, G.; Cemazar, M. Antitumor Effect of Antibiotic Resistance Gene-Free Plasmids Encoding Interleukin-12 in Canine Melanoma Model. **2018**, doi:10.1038/s41417-018-0014-5.

82. Landi, A.; Babiuk, L.A.; van Drunen Littel-van den Hurk, S. High Transfection Efficiency, Gene Expression, and Viability of Monocyte-Derived Human Dendritic Cells after Nonviral Gene Transfer. **2007**, *82*, doi:10.1189/jlb.0906561.

83. Laurent, V.; Fraix, A.; Montier, T.; Cammas-Marion, S.; Ribault, C.; Benvegnu, T.; Jaffres, P.-A.; Loyer, P. Highly Efficient Gene Transfer into Hepatocyte-like HepaRG Cells: New Means for Drug Metabolism and Toxicity Studies. **2010**, *5*, doi:10.1002/biot.200900255.

84. Lee, S.-L.S.-L.; Ock, S.-A.S.-A.; Yoo, J.-G.J.-G.; Kumar, B.M.M.; Choe, S.-Y.S.-Y.; Rho, G.-J.G.-J. Efficiency of Gene Transfection into Donor Cells for Nuclear Transfer of Bovine Embryos. **2005**, *72*, 191–200, doi:10.1002/mrd.20297.

85. Lehmann, M.H.; Berg, H. Electroporation Induced Gene Expression—a Case Study on Interleukin-10. *Bioelectrochemistry Bioenerg.* **1998**, *47*, 3–10, doi:10.1016/S0302-4598(98)00179-2.

86. Lewis, E.W.; Rudo, T.J.; St John, M.A.R.; Chu, J.L.; Heinze, A.W.; Howard, B.H.; Engleka, K.A. Endothelial Cell DNA Transfer and Expression Using Petri Dish Electroporation and the Nonreplicating Vaccinia Virus/T7 RNA Polymerase Hybrid System. **1999**, *6*, doi:10.1038/sj.gt.3300977.

87. Li, F.; Yamaguchi, K.; Okada, K.; Matsushita, K.; Enatsu, N.; Chiba, K.; Yue, H.; Fujisawa, M. Efficient Transfection of DNA into Primarily Cultured Rat Sertoli Cells by Electroporation1. **2013**, *88*, 61, doi:10.1095/biolreprod.112.106260.

88. Li, G.-B.; Lu, G.-X. Gene Delivery Efficiency in Bone Marrow-Derived Dendritic Cells: Comparison of Four Methods and Optimization for Lentivirus Transduction. *Mol. Biotechnol.* **2009**, *43*, 250–256, doi:10.1007/s12033-009-9197-1.

89. Li, J.; Yu, T.; Zhou, J.; Tu, W.; Gao, S.; Liu, X. Optimal Transfection Methods and Comparison of PK-15 and Dulac Cells for Rescue of Chimeric Porcine Circovirus Type 1-2. **2014**, *208*, doi:10.1016/j.jviromet.2014.08.005.

90. Liang, X.; Potter, J.; Kumar, S.; Zou, Y.; Quintanilla, R.; Sridharan, M.; Carte, J.; Chen, W.; Roark, N.; Ranganathan, S.; et al. Rapid and Highly Efficient Mammalian Cell Engineering via Cas9 Protein Transfection. **2015**, *208*, 44–53, doi:10.1016/j.jbiotec.2015.04.024.

91. Liew, A.; André, F.M.; Lesueur, L.L.; De Ménorval, M.-A.; O'Brien, T.; Mir, L.M. Robust, Efficient, and Practical Electrogene Transfer Method for Human Mesenchymal Stem Cells Using Square Electric Pulses. *Hum. Gene Ther. Methods* **2013**, *24*, 289–297, doi:10.1089/hgtb.2012.159.

92. Lin, W.-Z.; Lee, S.S.T.; Cheung, W.-T. Efficient Expression of Foreign Genes in CHO DHFR(-) Cells by Electroporation. *Biologicals* **2009**, *37*, 277–281, doi:10.1016/j.biologicals.2009.03.003.

93. Lin, Y.-C.; Jen, C.-M.; Huang, M.-Y.; Wu, C.-Y.; Lin, X.-Z. Electroporation Microchips for Continuous Gene Transfection. *Sensors Actuators B Chem.* **2001**, *79*, 137–143, doi:10.1016/S0925-4005(01)00859-0.

94. Lin, Y.-C.; Li, M.; Fan, C.-S.; Wu, L.-W. A Microchip for Electroporation of Primary Endothelial Cells. *Sensors Actuators A Phys.* **2003**, *108*, 12–19, doi:10.1016/j.sna.2003.05.002.

95. Lin, Y.-C.; Li, M.; Wu, C.-C. Simulation and Experimental Demonstration of the Electric Field Assisted Electroporation Microchip for in Vitro Gene Delivery Enhancement. **2004**, *4*, doi:10.1039/b312804k.

96. Liu, L.; Johnson, C.; Fujimura, S.; Teque, F.; Levy, J.A. Transfection Optimization for Primary Human CD8+ Cells. **2011**, *372*, doi:10.1016/j.jim.2011.06.026.

97. Liu, Y.; Bergan, R. Improved Intracellular Delivery of Oligonucleotides by Square Wave Electroporation. *Antisense Nucleic Acid Drug Dev.* **2001**, *11*, 7–14, doi:10.1089/108729001750072083.

98. Liu, Y.C.; Lin, W.Y.; Jhang, Y.R.; Huang, S.H.; Wu, C.P.; Wu, H.T. Efficiency of DNA Transfection of Rat Heart Myoblast Cells H9c2(2-1) by Either Polyethyleneimine or Electroporation. *Appl. Biochem. Biotechnol.* **2011**, *164*, 1172–1182, doi:10.1007/s12010-011-9203-5.

99. Lojk, J.; Mis, K.; Pirkmajer, S.; Pavlin, M. SiRNA Delivery into Cultured Primary Human Myoblasts--Optimization of Electroporation Parameters and Theoretical Analysis. *Bioelectromagnetics* **2015**, *36*, doi:10.1002/bem.21936.

100. Lundqvist, A.; Noffz, G.; Pavlenko, M.; Saebøe-Larssen, S.; Fong, T.; Maitland, N.; Pisa, P.; Sæbøe-Larssen, S.; Fong, T.; Maitland, N.; et al. Nonviral and Viral Gene Transfer Into Different Subsets of Human Dendritic Cells Yield Comparable Efficiency of Transfection. **2002**, *25*, doi:10.1097/00002371-200211000-00001.

101. Madeira, C.; Ribeiro, S.C.; Pinheiro, I.S.M.M.; Martins, S.A.M.M.; Andrade, P.Z.; da Silva, C.L.; Cabral, J.M.S.S. Gene Delivery to Human Bone Marrow Mesenchymal Stem Cells by Microporation. **2011**, *151*, doi:10.1016/j.jbiotec.2010.11.002.

102. Madeira, C.; Ribeiro, S.C.C.; Turk, M.Z.Z.; Cabral, J.M.S.M.S. Optimization of Gene Delivery to HEK293T Cells by Microporation Using a Central Composite Design Methodology. **2010**, *32*, doi:10.1007/s10529-010-0327-4.

103. Madeira, C.; Rodrigues, C.A. V; Reis, M.S.C.; Ferreira, F.F.C.G.; Correia, R.E.S.M.; Diogo, M.M.; Cabral, J.M.S. Nonviral Gene Delivery to Neural Stem Cells with Minicircles by Microporation. *Biomacromolecules* **2013**, *14*, doi:10.1021/bm400015b.

104. Marine, S.; Freeman, J.; Riccio, A.; Axenborg, M.-L.; Pihl, J.; Ketteler, R.; Aspengren, S. High-Throughput Transfection of Differentiated Primary Neurons from Rat Forebrain. *J. Biomol. Screen.* **2012**, *17*, 692–696, doi:10.1177/1087057112439233.

105. Marjanovič, I.; Haberl, S.S.; Miklavčič, D.; Kandušer, M.; Pavlin, M.; Marjanovic, I.; Haberl, S.S.; Miklavcic, D.; Kanduser, M.; Pavlin, M.; et al. Analysis and Comparison of Electrical Pulse Parameters for Gene Electrotransfer of Two Different Cell Lines. **2010**, *236*, 97–105, doi:10.1007/s00232-010-9282-1.

106. Marjanovič, I.; Kandušer, M.; Miklavčič, D.; Keber, M.M.M.M.; Pavlin, M. Comparison of Flow Cytometry, Fluorescence Microscopy and Spectrofluorometry for Analysis of Gene Electrotransfer Efficiency. **2014**, *247*, doi:10.1007/s00232-014-9714-4.

107. Markelc, B.; Tevz, G.; Cemazar, M.; Kranjc, S.; Lavrencak, J.; Zegura, B.; Teissie, J.; Sersa, G. Muscle Gene Electrotransfer Is Increased by the Antioxidant Tempol in Mice. *Gene Ther.* **2012**, *19*, 312–320, doi:10.1038/gt.2011.97.

108. Markowicz, S.; Niedzielska, J.; Kruszewski, M.; Ołdak, T.; Gajkowska, A.; Machaj, E.K.; Skurzak, H.; Pojda, Z. Nonviral Transfection of Human Umbilical Cord Blood Dendritic Cells Is Feasible, but the Yield of Dendritic Cells with Transgene Expression Limits the Application of This Method in Cancer Immunotherapy. *Acta Biochim. Pol.* **2006**, *53*, 203–212.

109. Mars, T.; Strazisar, M.; Mis, K.; Kotnik, N.; Pegan, K.; Lojk, J.; Grubic, Z.; Pavlin, M. Electrotransfection and Lipofection Show Comparable Efficiency for In Vitro Gene Delivery of Primary Human Myoblasts. **2015**, *248*, doi:10.1007/s00232-014-9766-5.

110. May, R.D.R.D.R.D.; Tekari, A.; Frauchiger, D.A.D.A.; Krismer, A.; Benneker, L.M.L.M.; Gantenbein, B. Efficient Nonviral Transfection of Primary Intervertebral Disc Cells by Electroporation for Tissue Engineering Application. **2017**, *23*, 30–37.

111. Mignet, N.; Vandermeulen, G.; Pembouong, G.; Largeau, C.; Thompson, B.; Spanedda, M.-V.; Wasungu, L.; Rols, M.-P.; Bessodes, M.; Bureau, M.F.; et al. Cationic and Anionic Lipoplexes Inhibit Gene Transfection by Electroporation in Vivo. *J. Gene Med.* **2010**, *12*, 491–500, doi:10.1002/jgm.1460.

112. Mohr, J.C.J.C.; de Pablo, J.J.J.J.; Palecek, S.P.S.P.; DePablo, J.J.; Palecek, S.P.S.P. Electroporation of Human Embryonic Stem Cells: Small and Macromolecule Loading and DNA Transfection. **2006**, *22*, doi:10.1021/bp0600334.

113. Müller, K.J.K.J.; Horbaschek, M.; Lucas, K.; Zimmermann, U.; Sukhorukov, V.L.V.L. Electrotransfection of Anchorage-Dependent Mammalian Cells. **2003**, *288*, doi:10.1016/S0014-4827(03)00224-6.

114. Mun, J.-Y.; Shin, K.K.; Kwon, O.; Lim, Y.T.; Oh, D.-B. Minicircle Microporation-Based Non-Viral Gene Delivery Improved the Targeting of Mesenchymal Stem Cells to an Injury Site. *Biomaterials* **2016**, *101*, doi:10.1016/j.biomaterials.2016.05.057.

115. Nakayama, A.; Sato, M.; Shinohara, M.; Matsubara, S.; Yokomine, T.; Akasaka, E.; Yoshida, M.; Takao, S. Efficient Transfection of Primarily Cultured Porcine Embryonic Fibroblasts Using the Amaxa Nucleofection System^{TM}. **2007**, *9*, doi:10.1089/clo.2007.0021.

116. Niakan, S.; Heidari, B.; Akbari, G.; Nikousefat, Z. Comparison of Different Electroporation Parameters on Transfection Efciency of Sheep Testicular Cells. *Cell J.* **2016**, *18*, 425–437.

117. Nickoloff, J.A.; Spirio, L.N.; Reynolds, R.J. A Comparison of Calcium Phosphate Coprecipitation and Electroporation. *Mol. Biotechnol.* **1998**, *10*, 93–101, doi:10.1007/BF02760857.

118. Ohse, M.; Tsuchida, K.; Tomita, H.; Taketo, A.; Kimoto, H.; Kusaoke, H. A New and Efficient Method for Gene Transfer into Mouse FM3A Cells Using Metaphase Chromosomes by Electroporation. *Biosci. Biotechnol. Biochem.* **1996**, *60*, 1879–1881, doi:10.1271/bbb.60.1879.

119. Ołdak, T.; Kruszewski, M.; Machaj, E.K.; Gajkowska, A.; Pojda, Z. Optimisation of Transfection Conditions of CD34+ Hematopoietic Cells Derived from Human Umbilical Cord Blood. *Acta Biochim. Pol.* **2002**, *49*, 625–632, doi:024903625.

120. Orio, J.; Bellard, E.; Baaziz, H.; Pichon, C.; Mouritzen, P.; Rols, M.-P.M.-P.; Teissié, J.; Golzio, M.; Chabot, S. Sub-Cellular Temporal and Spatial Distribution of Electrotransferred LNA/DNA Oligomer. **2013**, *9*.

121. Pasquet, L.; Bellard, E.; Rols, M.P.; Golzio, M.; Teissie, J. Post-Pulse Addition of Trans-Cyclohexane-1,2-Diol Improves Electrotransfer Mediated Gene Expression in Mammalian Cells. *Biochem. Biophys. Reports* **2016**, *7*, 287–294, doi:10.1016/j.bbrep.2016.07.012.

122. Pavlin, M.; Flisar, K.; Kandušer, M.; Kanduser, M.; Kandušer, M.; Kanduser, M. The Role of Electrophoresis in Gene Electrotransfer. **2010**, *236*, doi:10.1007/s00232-010-9276-z.

123. Pavlin, M.; Kandušer, M. New Insights into the Mechanisms of Gene Electrotransfer - Experimental and Theoretical Analysis. *Sci. Rep.* **2015**, *5*, doi:10.1038/srep09132.

124. Pelofy, S.; Teissié, J.; Golzio, M.; Chabot, S. Chemically Modified Oligonucleotide–Increased Stability Negatively Correlates with Its Efficacy Despite Efficient Electrotransfer. **2012**, *245*, 565–571, doi:10.1007/s00232-012-9468-9.

125. Peng, H.; Wu, Y.; Zhang, Y. Efficient Delivery of DNA and Morpholinos into Mouse Preimplantation Embryos by Electroporation. **2012**, *7*, e43748, doi:10.1371/journal.pone.0043748.

126. Peng, L.; Xiong, W.; Cai, Y.; Chen, Y.; He, Y.; Yang, J.; Jin, J.; Li, H. A Simple, Rapid Method for Evaluation of Transfection Efficiency Based on Fluorescent Dye. *Bioengineered* **2017**, *8*, 225–231, doi:10.1080/21655979.2016.1222995.

127. Prosen, L.; Markelc, B.; Dolinsek, T.; Music, B.; Cemazar, M.; Sersa, G. Mcam Silencing With RNA Interference Using Magnetofection Has Antitumor Effect in Murine Melanoma. **2014**, *3*, e205, doi:10.1038/mtna.2014.56.

128. Rebersek, M.; Kanduser, M.; Miklavcic, D. Pipette Tip with Integrated Electrodes for Gene Electrotransfer of Cells in Suspension: A Feasibility Study in CHO Cells. **2011**, *45*, doi:10.2478/v10019-011-0025-4.

129. Regnier, V.; Le Doan, T.; Préat, V. Parameters Controlling Topical Delivery of Oligonucleotides by Electroporation. *J. Drug Target.* **1998**, *5*, 275–289, doi:10.3109/10611869808995881.

130. Rizk, A.; Rabie, B.M. Electroporation for Transfection and Differentiation of Dental Pulp Stem Cells. **2013**, *2*, doi:10.1089/biores.2012.0273.

131. ROLS, M.-P.; COULET, D.; TEISSIE, J. Highly Efficient Transfection of Mammalian Cells by Electric Field Pulses. Application to Large Volumes of Cell Culture by Using a Flow System. *Eur. J. Biochem.* **1992**, *206*, 115–121, doi:10.1111/j.1432-1033.1992.tb16908.x.

132. Rols, M.-P.; Delteil, C.; Golzio, M.; Teissié, J. In Vitro and Ex Vivo Electrically Mediated

Permeabilization and Gene Transfer in Murine Melanoma. *Bioelectrochemistry Bioenerg.* **1998**, *47*, 129–134, doi:10.1016/S0302-4598(98)00181-0.

133. Ross, J.W.; Whyte, J.J.; Zhao, J.; Samuel, M.; Wells, K.D.; Prather, R.S. Optimization of Square-Wave Electroporation for Transfection of Porcine Fetal Fibroblasts. *Transgenic Res.* **2010**, *19*, 611–620, doi:10.1007/s11248-009-9345-1.

134. Ruybal, P.; Gravisaco, M.J.; Barcala, V.; Escalada, A.; Cremaschi, G.; Taboga, O.; Waldner, C.; Mongini, C. Transgene Expression Enhancement in T-Lymphoma Cell Lines. *Int. Immunopharmacol.* **2005**, *5*, 1685–1692, doi:10.1016/j.intimp.2005.04.016.

135. Salimzadeh, L.; Jaberipour, M.; Hosseini, A.; Ghaderi, A. Non-Viral Transfection Methods Optimized for Gene Delivery to a Lung Cancer Cell Line. *Avicenna J. Med. Biotechnol.* **2013**, *5*, 68–77.

136. Satyabhama, S.; Epstein, A.L. Short-Term Efficient Expression of Transfected DNA in Human Hematopoietic Cells by Electroporation: Definition of Parameters and Use of Chemical Stimulators. *DNA* **1988**, *7*, 203–209, doi:10.1089/dna.1988.7.203.

137. Schakowski, F.; Buttgereit, P.; Mazur, M.; Märten, A.; Schöttker, B.; Gorschlüter, M.; Schmidt-Wolf, I.G.H. Novel Non-Viral Method for Transfection of Primary Leukemia Cells and Cell Lines. *Genet. Vaccines Ther.* **2004**, *2*, doi:10.1186/1479-0556-2-1.

138. Schakowski, F.; Gorschlüter, M.; Junghans, C.; Schroff, M.; Buttgereit, P.; Ziske, C.; Schöttker, B.; König-Merediz, S.A.; Sauerbruch, T.; Wittig, B.; et al. A Novel Minimal-Size Vector (MIDGE) Improves Transgene Expression in Colon Carcinoma Cells and Avoids Transfection of Undesired DNA. **2001**, *3*, 793–800, doi:10.1006/mthe.2001.0322.

139. Seo, H.W.; Kim, T.M.; Choi, J.W.; Han, B.K.; Song, G.; Han, J.Y. Evaluation of Combinatorial Cis-Regulatory Elements for Stable Gene Expression in Chicken Cells. *BMC Biotechnol.* **2010**, *10*, 69, doi:10.1186/1472-6750-10-69.

140. Smirnikhina, S.A.; Lavrov, A. V; Bochkov, N.P. Dynamics of Elimination of Plasmids and Expression of VEGF121 Gene Transfected into Human Mesenchymal Stem Cells by Different Methods. *Bull. Exp. Biol. Med.* **2011**, *151*, 121–125, doi:10.1007/s10517-011-1272-5.

141. Spandidos, D.A.D.A. Electric Field-Mediated Gene Transfer (Electroporation) into Mouse Friend and Human K562 Erythroleukemic Cells. **1987**, *4*, doi:10.1016/0735-0651(87)90018-5.

142. Stacey, K.J.; ROSS, I.A.N.L.; HUME, D.A. Electroporation and DNA-Dependent Cell Death in Murine Macrophages. **1993**, 75–85, doi:10.1038/icb.1993.8.

143. Stopper, H.; Jones, H.; Zimmermann, U. Large Scale Transfection of Mouse L-Cells by Electropermeabilization. *BBA - Biomembr.* **1987**, *900*, 38–44, doi:10.1016/0005-2736(87)90275-6.

144. Stopper, H.; Zimmermann, U.; Neil, G.A. Increased Efficiency of Transfection of Murine Hybridoma Cells with DNA by Electropermeabilization. *J. Immunol. Methods* **1988**, *109*, 145–151, doi:10.1016/0022-1759(88)90452-8.

145. Stopper, H.; Zimmermann, U.; Wecker, E. High Yields of DNA-Transfer Into Mouse L-Cells by Electropermeabilization. *Zeitschrift fur Naturforsch. - Sect. C J. Biosci.* **1985**, *40*, 929–932, doi:10.1515/znc-1985-11-1233.

146. Stroh, T.; Erben, U.; Kühl, A.A.A.A.; Zeitz, M.; Siegmund, B. Combined Pulse Electroporation – A Novel Strategy for Highly Efficient Transfection of Human and Mouse Cells. **2010**, *5*, e9488, doi:10.1371/journal.pone.0009488.

147. Sukharev, S.I.; Klenchin, V.A.; Serov, S.M.; Chernomordik, L. V; Chizmadzhev YuA Electroporation and Electrophoretic DNA Transfer into Cells. The Effect of DNA Interaction with Electropores. *Biophys. J.* **1992**, *63*, 1320–1327, doi:10.1016/S0006-3495(92)81709-5.

148. Sungailaitė, S.; Ruzgys, P.; Šatkauskienė, I.; Čepurnienė, K.; Šatkauskas, S. The Dependence of Efficiency of Transmembrane Molecular Transfer Using Electroporation on Medium Viscosity. *J. Gene Med.* **2015**, *17*, 80–86, doi:10.1002/jgm.2825.

149. Tabar, M.S.; Hesaraki, M.; Esfandiari, F.; Samani, F.S.; Vakilian, H.; Baharvand, H.; Sharifi Tabar, M.; Hesaraki, M.; Esfandiari, F.; Sahraneshin Samani, F.; et al. Evaluating Electroporation and Lipofectamine Approaches for Transient and Stable Transgene Expressions in Human Fibroblasts and Embryonic Stem Cells. **2015**, *17*.

150. Takahashi, M.; Furukawa, T.; Saitoh, H.; Aoki, A.; Koike, T.; Moriyama, Y.; Shibata, A. Gene Transfer into Human Leukemia Cell Lines by Electroporation: Experience with Exponentially Decaying and Square Wave Pulse. *Leuk. Res.* **1991**, *15*, 507–513, doi:10.1016/0145-2126(91)90062-X.

151. Tanaka, M.; Yanagawa, Y.; Hirashima, N. Transfer of Small Interfering RNA by Single-Cell Electroporation in Cerebellar Cell Cultures. **2009**, *178*, doi:10.1016/j.jneumeth.2008.11.025.

152. Taylor, W.; Gokay, K.E.; Capaccio, C.; Davis, E.; Glucksberg, M.; Dean, D.A. The Effects of Cyclic Stretch on Gene Transfer in Alveolar Epithelial Cells. **2003**, *7*, doi:10.1016/S1525-0016(03)00041-8.

153. Teifel, M.; Heine, L.-T.; Milbredt, S.; Friedl, P. Optimization of Transfection of Human Endothelial Cells. *Endothelium* **1997**, *5*, 21–35, doi:10.3109/10623329709044156.

154. Tekle, E.; Astumian, R.D.; Chock, P.B. Electroporation by Using Bipolar Oscillating Electric Field: An Improved Method for DNA Transfection of NIH 3T3 Cells. **1991**, *88*, doi:10.1073/pnas.88.10.4230.

155. Tesic, N.; Cemazar, M. In Vitro Targeted Gene Electrotransfer to Endothelial Cells with Plasmid DNA Containing Human Endothelin-1 Promoter. **2013**, *246*, doi:10.1007/s00232-013-9548-5.

156. Tur-Kaspa, R.; Teicher, L.; Levine, B.J.; Skoultchi, A.I.; Shafritz, D.A. Use of Electroporation to Introduce Biologically Active Foreign Genes into Primary Rat Hepatocytes. *Mol. Cell. Biol.* **1986**, *6*, 716–718.

157. Underhill, M.F.; Coley, C.; Birch, J.R.; Findlay, A.; Kallmeier, R.; Proud, C.G.; James, D.C. Engineering MRNA Translation Initiation to Enhance Transient Gene Expression in Chinese Hamster Ovary Cells. *Biotechnol. Prog.* **2003**, *19*, 121–129, doi:10.1021/bp025560b.

158. Van Gaal, E.V.B.; Oosting, R.S.; Van Eijk, R.; Bakowska, M.; Feyen, D.; Kok, R.J.; Hennink, W.E.; Crommelin, D.J.A.; Mastrobattista, E. DNA Nuclear Targeting Sequences for Non-Viral Gene Delivery. *Pharm. Res.* **2011**, *28*, 1707–1722, doi:10.1007/s11095-011-0407-8.

159. van Leeuwen, E.B.M.B.; van der Veen, A.Y.Y.; Hoekstra, D.; Engberts, J.B.F.N.B.; Halie, M.R.R.; Van Der Meer, J.; Ruiters, M.H.J.H. Transfection of Small Numbers of Human Endothelial Cells by

Electroporation and Synthetic Amphiphiles. **1999**, *17*, 9–14, doi:10.1053/ejvs.1998.0677.

160. Van Meirvenne, S.; Straetman, L.; Heirman, C.; Dullaers, M.; De Greef, C.; Van Tendeloo, V.; Thielemans, K. Efficient Genetic Modification of Murine Dendritic Cells by Electroporation with MRNA. **2002**, *9*, doi:10.1038/sj.cgt.7700499.

161. Van Tendeloo, V.F.I.; Ponsaerts, P.; Lardon, F.; Nijs, G.; Lenjou, M.; Van Broeckhoven, C.; Van Bockstaele, D.R.; Berneman, Z.N. Highly Efficient Gene Delivery by MRNA Electroporation in Human Hematopoietic Cells: Superiority to Lipofection and Passive Pulsing of MRNA and to Electroporation of Plasmid CDNA for Tumor Antigen Loading of Dendritic Cells. *Blood* **2001**, *98*, 49–56, doi:10.1182/blood.V98.1.49.

162. Wang, S.; Zhang, X.; Wang, W.; Lee, L.J. Semicontinuous Flow Electroporation Chip for High-Throughput Transfection on Mammalian Cells. **2009**, *81*, doi:10.1021/ac9002672.

163. Wasungu, L.; Escoffre, J.-M.; Valette, A.; Teissie, J.; Rols, M.-P. A 3D in Vitro Spheroid Model as a Way to Study the Mechanisms of Electroporation. *Int. J. Pharm.* **2009**, *379*, 278–284, doi:10.1016/j.ijpharm.2009.03.035.

164. Wasungu, L.; Pillet, F.; Bellard, E.; Rols, M.-P.; Teissié, J. Shock Waves Associated with Electric Pulses Affect Cell Electro-Permeabilization. **2014**, *100*, 36–43, doi:10.1016/j.bioelechem.2014.06.011.

165. Wawrzynska, M.; Bednarczyk, M.; Łakota, P.; Lubiszewska, M. Influence of Electroporation on Chicken Blastoderm Cell Viability in Vitro. *Folia Biol. (Praha).* **2008**, *56*, 197–201, doi:10.3409/fb.56_3-4.197-201.

166. Wei, Z.; Li, X.; Zhao, D.; Yan, H.; Hu, Z.; Liang, Z.; Li, Z. Flow-Through Cell Electroporation Microchip Integrating Dielectrophoretic Viable Cell Sorting. **2014**, *86*, doi:10.1021/ac502294e.

167. Wei, Z.; Zhao, D.; Li, X.; Wu, M.; Wang, W.; Huang, H.; Wang, X.; Du, Q.; Liang, Z.; Li, Z. A Laminar Flow Electroporation System for Efficient DNA and SiRNA Delivery. **2011**, *83*, doi:10.1021/ac200625b.

168. Wei, Z.-L.; Ogawa, R.; Takasaki, I.; Zhao, Q.-L.; Zheng, H.-C.; Ahmed, K.; Hassan, M.A.; Kondo, T. Mild Hyperthermia Prior to Electroporation Increases Transfection Efficiency in HCT 116, HeLa S3 and SGC 7901 Cells. *Biotechnol. Lett.* **2010**, *32*, 367–371, doi:10.1007/s10529-009-0164-5.

169. Wells, J.M.M.; Li, L.H.H.; Sen, A.; Jahreis, G.P.P.; Hui, S.W.W. Electroporation-Enhanced Gene Delivery in Mammary Tumors. **2000**, *7*, doi:10.1038/sj.gt.3301141.

170. Will, A.; Röllinghoff, M.; Gessner, A. Stable Transfection of Cloned Murine T Helper Cells. *J. Immunol. Methods* **1995**, *188*, 139–146, doi:10.1016/0022-1759(95)00212-X.

171. Wolf, H.; Rols, M.P.; Boldt, E.; Neumann, E.; Teissié, J. Control by Pulse Parameters of Electric Field-Mediated Gene Transfer in Mammalian Cells. *Biophys. J.* **1994**, *66*, 524–531.

172. Wu, M.; Yuan, F. Membrane Binding of Plasmid DNA and Endocytic Pathways Are Involved in Electrotransfection of Mammalian Cells. *PLoS One* **2011**, *6*, e20923, doi:10.1371/journal.pone.0020923.

173. Xu, Y.; Lu, Y.; Xing, W. An Individually Addressable Suspended-Drop Electroporation System for High-Throughput Cell Transfection. *Lab Chip* **2014**, *14*, 686–690, doi:10.1039/c3lc50907a.

174. Xu, Y.; Su, S.; Zhou, C.; Lu, Y.; Xing, W. Cell Electroporation with a Three-Dimensional Microelectrode Array on a Printed Circuit Board. **2015**, *102*, 35–41, doi:10.1016/j.bioelechem.2014.10.002.

175. Yang, S.-C.; Huang, K.-S.; Chen, H.-Y.; Lin, Y.-C. Determination of Optimum Gene Transfection Conditions Using the Taguchi Method for an Electroporation Microchip. *Sensors Actuators, B Chem.* **2008**, *132*, 551–557, doi:10.1016/j.snb.2007.11.037.

176. Yao, S.; Rana, S.; Liu, D.; Wise, G.E.G.E. Electroporation Optimization to Deliver Plasmid DNA into Dental Follicle Cells. **2009**, *4*, doi:10.1002/biot.200900039.

177. Yu, L.; Reynaud, F.; Falk, J.; Spencer, A.; Ding, Y.-D.Y.-D.; BaumlÃ©, V.; Lu, R.; Castellani, V.; Rudkin, B.B.B.B.; Yuan, C.; et al. Highly Efficient Method for Gene Delivery into Mouse Dorsal Root Ganglia Neurons. *Front. Mol. Neurosci.* **2015**, *8*, doi:10.3389/fnmol.2015.00002.

178. Zhang, Z.; Qiu, S.; Zhang, X.; Chen, W. Optimized DNA Electroporation for Primary Human T Cell Engineering. **2018**, *18*, 4, doi:10.1186/s12896-018-0419-0.

179. Zhou, R.; Norton, J.E.; Zhang, N.; Dean, D.A. Electroporation-Mediated Transfer of Plasmids to the Lung Results in Reduced TLR9 Signaling and Inflammation. **2007**, *14*, doi:10.1038/sj.gt.3302936.

180. Zizzi, A.; Minardi, D.; Ciavattini, A.; Giantomassi, F.; Montironi, R.; Muzzonigro, G.; Di Primio, R.; Lucarini, G. Green Fluorescent Protein as Indicator of Nonviral Transient Transfection Efficiency in Endometrial and Testicular Biopsies. *73*, doi:10.1002/jemt.20779.

181. Znidar, K.; Bosnjak, M.; Semenova, N.; Pakhomova, O.; Heller, L.; Cemazar, M. Tumor Cell Death after Electrotransfer of Plasmid DNA Is Associated with Cytosolic DNA Sensor Upregulation. *Oncotarget* **2018**, *9*, 18665–18681, doi:10.18632/oncotarget.24816.

182. Haberl, S.; Kandušer, M.; Flisar, K.; Hodžić, D.; Bregar, V.B.V.B.; Miklavčič, D.; Escoffre, J..-M.J.-M.; Rols, M.-P.M..-P.; Pavlin, M. Effect of Different Parameters Used for in Vitro Gene Electrotransfer on Gene Expression Efficiency, Cell Viability and Visualization of Plasmid DNA at the Membrane Level. **2013**, *15*, doi:10.1002/jgm.2706.

183. Sieni, E.; Dettin, M.; De Robertis, M.; Bazzolo, B.; Conconi, M.T.; Zamuner, A.; Marino, R.; Keller, F.; Campana, L.G.; Signori, E. The Efficiency of Gene Electrotransfer in Breast-Cancer Cell Lines Cultured on a Novel Collagen-Free 3D Scaffold. *Cancers (Basel).* **2020**, *12*, doi:10.3390/cancers12041043.

184. Pinyon, J.L.; Klugmann, M.; Lovell, N.H.; Housley, G.D. Dual-Plasmid Bionic Array-Directed Gene Electrotransfer in HEK293 Cells and Cochlear Mesenchymal Cells Probes Transgene Expression and Cell Fate. *Hum. Gene Ther.* **2019**, *30*, 211–224, doi:10.1089/hum.2018.062.

185. Salkin, H.; Gönen, Z.B.; Ergen, E.; Bahar, D.; Çetin, M. Effects of TGF-$β$1 Overexpression on Biological Characteristics of Human Dental Pulp-Derived Mesenchymal Stromal Cells. *Int. J. Stem Cells* **2019**, *12*, 170–182, doi:10.15283/ijsc18051.

186. Hyder, I.; Eghbalsaied, S.; Kues, W.A. Systematic Optimization of Square-Wave Electroporation Conditions for Bovine Primary Fibroblasts. *BMC Mol. Cell Biol.* **2020**, *21*, doi:10.1186/s12860-020-00254-5.

187. Ruzgys, P.; Novickij, V.; Novickij, J.; Šatkauskas, S. Nanosecond Range Electric Pulse Application as a Non-Viral Gene Delivery Method: Proof of Concept. *Sci. Rep.* **2018**, *8*, doi:10.1038/s41598-018-

33912-y.

188. Sherba, J.J.; Hogquist, S.; Lin, H.; Shan, J.W.; Shreiber, D.I.; Zahn, J.D. The Effects of Electroporation Buffer Composition on Cell Viability and Electro-Transfection Efficiency. *Sci. Rep.* **2020**, *10*, doi:10.1038/s41598-020-59790-x.

189. Chopra, S.; Ruzgys, P.; Maciulevičius, M.; Jakutavičiute, M.; Šatka, S. Investigation of Plasmid DNA Delivery and Cell Viability Dynamics for Optimal Cell Electrotransfection in Vitro. *Appl. Sci.* **2020**, *10*, doi:10.3390/app10176070.

190. Heller, L.; Bulysheva, A.; Arpag, S.; Sales Conniff, A.; Kohena, K.; Shi, G.; Semenova, N.; Heller, R.; Cemazar, M. Growth Environment Influences B16.F10 Mouse Melanoma Cell Response to Gene Electrotransfer. *Bioelectrochemistry* **2021**, *140*, 107827, doi:10.1016/j.bioelechem.2021.107827.

191. Potočnik, T.; Miklavčič, D.; Maček Lebar, A. Gene Transfer by Electroporation with High Frequency Bipolar Pulses in Vitro. *Bioelectrochemistry* **2021**, 107803, doi:10.1016/j.bioelechem.2021.107803.

192. Keyer, V. V; Shevtsov, A.B.; Zaripov, M.M.; Baltabekova, A.Z.; Ramanculov, E.M.; Shustov, A. V Towards Development of Plasmacytoma Cells-Based Expression Systems Utilizing Alphavirus Vectors: An NS0-VEE Model. *J. Virol. Methods* **2019**, *274*, doi:10.1016/j.jviromet.2019.113734.

193. Lissandrello, C.A.; Santos, J.A.; Hsi, P.; Welch, M.; Mott, V.L.; Kim, E.S.; Chesin, J.; Haroutunian, N.J.; Stoddard, A.G.; Czarnecki, A.; et al. High-Throughput Continuous-Flow Microfluidic Electroporation of MRNA into Primary Human T Cells for Applications in Cellular Therapy Manufacturing. *Sci. Rep.* **2020**, *10*, doi:10.1038/s41598-020-73755-0.

194. Tereshchenko, V.; Bulygin, A.; Zavodskii, R.; Maksyutov, A.; Kurilin, V.; Fisher, M.; Semenyuk, N.; Aladev, S.; Sennikov, S. The Murine DCs Transfected with DNA-Plasmid Encoding CCR9 Demonstrate the Increased Migration to CCL25 and Thymic Cells in Vitro and to the Thymus in Vivo. *Cytokine* **2021**, *142*, doi:10.1016/j.cyto.2021.155473.

195. Zu, Y.; Liu, X.; Chang, A.Y.; Wang, S. Flow Micropillar Array Electroporation to Enhance Size Specific Transfection to a Large Population of Cells. *Bioelectrochemistry* **2020**, *132*, doi:10.1016/j.bioelechem.2019.107417.

196. Campillo-Davo, D.; Fujiki, F.; Van Den Bergh, J.M.J.; De Reu, H.; Smits, E.L.J.M.; Goossens, H.; Sugiyama, H.; Lion, E.; Berneman, Z.N.; Van Tendeloo, V. Efficient and Non-Genotoxic RNA-Based Engineering of Human T Cells Using Tumor-Specific t Cell Receptors with Minimal TCR Mispairing. *Front. Immunol.* **2018**, *9*, doi:10.3389/fimmu.2018.02503.

197. Yudovich, D.; Bäckström, A.; Schmiderer, L.; Žemaitis, K.; Subramaniam, A.; Larsson, J. Combined Lentiviral- and RNA-Mediated CRISPR/Cas9 Delivery for Efficient and Traceable Gene Editing in Human Hematopoietic Stem and Progenitor Cells. *Sci. Rep.* **2020**, *10*, doi:10.1038/s41598-020-79724-x.

198. Von Der Haar, K.; Jonczyk, R.; Lavrentieva, A.; Weyand, B.; Vogt, P.; Jochums, A.; Stahl, F.; Scheper, T.; Blume, C.A. Electroporation: A Sustainable and Cell Biology Preserving Cell Labeling Method for Adipogenous Mesenchymal Stem Cells. *Biores. Open Access* **2019**, *8*, 32–44, doi:10.1089/biores.2019.0001.

199. Nowaczyk, M.; Malcher, A.; Zimna, A.; Łabędź, W.; Kubaszewski, Ł.; Fiedorowicz, K.; Wierzbiński, K.; Rozwadowska, N.; Kurpisz, M. Transient and Stable Overexpression of Extracellular Superoxide

Dismutase Is Positively Associated with the Myogenic Function of Human Skeletal Muscle-Derived Stem/Progenitor Cells. *Antioxidants* **2020**, *9*, 1–19, doi:10.3390/antiox9090817.

200. Yu, X.; Fang, X.; Xiao, H.; Zhao, Z.; Maak, S.; Wang, M.; Yang, R. The Effect of Acyl-CoA Synthetase Long-Chain Family Member 5 on Triglyceride Synthesis in Bovine Preadipocytes. *Arch. Anim. Breed.* **2019**, *62*, 257–264, doi:10.5194/aab-62-257-2019.

201. Gaebler, A.M.; Hennig, A.; Buczolich, K.; Weitz, J.; Welsch, T.; Stange, D.E.; Pape, K. Universal and Efficient Electroporation Protocol for Genetic Engineering of Gastrointestinal Organoids. *J. Vis. Exp.* **2020**, *2020*, doi:10.3791/60704.

202. Liu, X.; Zu, Y.; Wang, S. Cell Size-Specific Transfection by Micropillar Array Electroporation. In *Methods in Molecular Biology*; Humana Press Inc., 2020; Vol. 2050, pp. 3–12.

203. Ishino, T.; Hashimoto, M.; Amagasa, M.; Saito, N.; Dochi, O.; Kirisawa, R.; Kitamura, H. Establishment of Protocol for Preparation of Gene-Edited Bovine Ear-Derived Fibroblasts for Somatic Cell Nuclear Transplantation. *Biomed. Res.* **2018**, *39*, 95–104, doi:10.2220/biomedres.39.95.

204. Kopru, C.Z.; Cagnan, I.; Akar, I.; Esendagli, G.; Korkusuz, P.; Gunel-Ozcan, A. Dual Effect of Glucocorticoid-Induced Tumor Necrosis Factor–Related Receptor Ligand Carrying Mesenchymal Stromal Cells on Small Cell Lung Cancer: A Preliminary in Vitro Study. *Cytotherapy* **2018**, *20*, 930–940, doi:10.1016/j.jcyt.2018.05.002.

205. Zhou, Y.; Lu, Y.; Cheng, J.; Xu, Y. Highly Uniform In-Situ Cell Electrotransfection of Adherent Cultures Using Grouped Interdigitated Electrodes. *Bioelectrochemistry* **2020**, *132*, doi:10.1016/j.bioelechem.2019.107435.

206. Sonam Chopra; Ruzgys, P.; Maciulevičius, M.; Šatkauskas, S. Effect of Cell Passage Time on the Electrotransfection Efficiency. *Biol. Bull.* **2020**, *47*, 441–447, doi:10.1134/S1062359020550014.

207. Stolwijk, J.A.; Wegener, J. Impedance Analysis of Adherent Cells after in Situ Electroporation-Mediated Delivery of Bioactive Proteins, DNA and Nanoparticles in ML-Volumes. *Sci. Rep.* **2020**, *10*, doi:10.1038/s41598-020-78096-6.

208. Zhu, Q.; Hamilton, M.; Vasquez, B.; He, M. 3D-Printing Enabled Micro-Assembly of a Microfluidic Electroporation System for 3D Tissue Engineering. *Lab Chip* **2019**, *19*, 2362–2372, doi:10.1039/c9lc00046a.

209. Potočnik, T.; Miklavčič, D.; Maček Lebar, A. Effect of Electroporation and Recovery Medium PH on Cell Membrane Permeabilization, Cell Survival and Gene Transfer Efficiency in Vitro. *Bioelectrochemistry* **2019**, *130*, doi:10.1016/j.bioelechem.2019.107342.

210. Ishino, T.; Kurita, H.; Kirisawa, R.; Shimamoto, Y.; Numano, R.; Kitamura, H. Introduction of a Plasmid and a Protein into Bovine and Swine Cells by Water-in-Oil Droplet Electroporation. *J. Vet. Med. Sci.* **2020**, *82*, 14–22, doi:10.1292/jvms.19-0475.

211. Campillo-Davo, D.; Versteven, M.; Roex, G.; De Reu, H.; van der Heijden, S.; Anguille, S.; Berneman, Z.N.; Van Tendeloo, V.F.I.; Lion, E. Rapid Assessment of Functional Avidity of Tumor-Specific T Cell Receptors Using an Antigen-Presenting Tumor Cell Line Electroporated with Full-Length Tumor Antigen MRNA. *Cancers (Basel).* **2020**, *12*, doi:10.3390/cancers12020256.

212. Wang, L.; Chang, C.C.; Sylvers, J.; Yuan, F. A Statistical Framework for Determination of Minimal

Plasmid Copy Number Required for Transgene Expression in Mammalian Cells. *Bioelectrochemistry* **2021**, *138*, doi:10.1016/j.bioelechem.2020.107731.

213. Bruter, A. V; Kandarakov, O.F.; Belyavsky, A. V Persistence of Plasmid-Mediated Expression of Transgenes in Human Mesenchymal Stem Cells Depends Primarily on CpG Levels of Both Vector and Transgene. *J. Gene Med.* **2018**, *20*, e3009, doi:10.1002/jgm.3009.

214. Zhang, Y.; Yin, C.; Hu, L.; Chen, Z.; Zhao, F.; Li, D.; Ma, J.; Ma, X.; Su, P.; Qiu, W.; et al. MACF1 Overexpression by Transfecting the 21 Kbp Large Plasmid PEGFP-C1A-ACF7 Promotes Osteoblast Differentiation and Bone Formation. *Hum. Gene Ther.* **2018**, *29*, 259–270, doi:10.1089/hum.2017.153.

215. Aghvami, T.A.; Latifi-Navid, S.; Zahri, S.; Sagha, M. Optimization of PCMV3-Nog-C-GFPSpark Electro-Transfection in MCF-7, a Breast Cancer Cell Line. *Res. J. Biotechnol.* **2021**, *16*, 13–18, doi:10.25303/1610RJBT1318.

216. Komel, T.; Bosnjak, M.; Kranjc Brezar, S.; De Robertis, M.; Mastrodonato, M.; Scillitani, G.; Pesole, G.; Signori, E.; Sersa, G.; Cemazar, M. Gene Electrotransfer of IL-2 and IL-12 Plasmids Effectively Eradicated Murine B16.F10 Melanoma. *Bioelectrochemistry* **2021**, *141*, doi:10.1016/J.BIOELECHEM.2021.107843.

217. Radzevičiūtė, E.; Malyško-Ptašinskė, V.; Novickij, J.; Novickij, V.; Girkontaitė, I. Transfection by Electroporation of Cancer and Primary Cells Using Nanosecond and Microsecond Electric Fields. *Pharmaceutics* **2022**, *14*, 1239, doi:10.3390/PHARMACEUTICS14061239.

218. Eghbalsaied, S.; Kues, W.A. An Electrochemical Protocol for CRISPR-Mediated Gene-Editing of Sheep Embryonic Fibroblast Cells. *Cells. Tissues. Organs* **2021**, doi:10.1159/000521128.

219. Bosnjak, M.; Znidar, K.; Sales Conniff, A.; Jesenko, T.; Markelc, B.; Semenova, N.; Tur, J.; Kohena, K.; Kranjc Brezar, S.; Heller, L.; et al. In Vitro and in Vivo Correlation of Skin and Cellular Responses to Nucleic Acid Delivery. *Biomed. Pharmacother.* **2022**, *150*, doi:10.1016/J.BIOPHA.2022.113088.

220. Hun, T.; Zhang, Y.; Xu, Q.; Huang, D.; Wang, Q.; Li, Z.; Wang, W. In Situ Electroporation on PERFECT Filter for High-Efficiency and High-Viability Tumor Cell Labeling. *Micromachines* **2022**, *13*, doi:10.3390/MI13050672.

221. Patino, C.A.; Mukherjee, P.; Berns, E.J.; Moully, E.H.; Stan, L.; Mrksich, M.; Espinosa, H.D. High-Throughput Microfluidics Platform for Intracellular Delivery and Sampling of Biomolecules from Live Cells. *ACS Nano* **2022**, doi:10.1021/ACSNANO.2C00698.

222. Nakami, W.N.; Nguhiu-Mwangi, J.; Kipyegon, A.N.; Ogugo, M.; Muteti, C.; Kemp, S. Comparative Efficiency for in Vitro Transfection of Goat Undifferentiated Spermatogonia Using Lipofectamine Reagents and Electroporation. *Stem Cells Cloning Adv. Appl.* **2022**, *Volume 15*, 11–20, doi:10.2147/SCCAA.S356588.

223. Radzevičiūtė, E.; Murauskas, A.; Ruzgys, P.; Šatkauskas, S.; Girkontaitė, I.; Novickij, J.; Novickij, V. Dielectrophoretic Manipulation of Cell Transfection Efficiency during Electroporation Using a Center Needle Electrode. *Appl. Sci.* **2021**, *11*, doi:10.3390/app11157015.

224. Kardani, K.; Milani, A.; Bolhassani, A. Gene Delivery in Adherent and Suspension Cells Using the Combined Physical Methods. *Cytotechnology* **2022**, *74*, 245–257, doi:10.1007/S10616-022-00524-4.

225. Tabak, S.; Feinshtein, V.; Schreiber-Avissar, S.; Beit-Yannai, E. Non-Pigmented Ciliary Epithelium-Derived Extracellular Vesicles Loaded with Smad7 Sirna Attenuate Wnt Signaling in Trabecular Meshwork Cells in Vitro. *Pharmaceuticals* **2021**, *14*, doi:10.3390/PH14090858.

226. Bulygin, A.S.; Tereshchenko, V.P.; Zavodskii, R.Y.; Obleukhova, I.A.; Sennikov, S. V; Silkov, A.N. Effect of DNA Constructions Electroporation on Dendritic Cells. *Med. Immunol.* **2021**, *23*, 653–658, doi:10.15789/1563-0625-EOD-2243.

227. Kanazawa, T.; Hoashi, Y.; Ibaraki, H.; Takashima, Y.; Okada, H. Electroporation-Based Ex Vivo Gene Delivery into Dendritic Cells by Anionic Polymer-Coated Versatile Nuclear Localization Signal/PDNA Complex. *Biol. Pharm. Bull.* **2021**, *44*, 1866–1871, doi:10.1248/BPB.B21-00559.

228. Pelofy, S.; Bousquet, H.; Gibot, L.; Rols, M.P.; Golzio, M. Transfer of Small Interfering RNA by Electropermeabilization in Tumor Spheroids. *Bioelectrochemistry* **2021**, *141*, 107848, doi:10.1016/j.bioelechem.2021.107848.
